# A prospective longitudinal study on the microbiota composition in amyotrophic lateral sclerosis

**DOI:** 10.1186/s12916-020-01607-9

**Published:** 2020-06-17

**Authors:** Diana Di Gioia, Nicole Bozzi Cionci, Loredana Baffoni, Angela Amoruso, Marco Pane, Luca Mogna, Francesca Gaggìa, Maria Ausiliatrice Lucenti, Enrica Bersano, Roberto Cantello, Fabiola De Marchi, Letizia Mazzini

**Affiliations:** 1 Department of Agricultural and Food Sciences, University of Bologna, Viale Fanin 42, Bologna, Italy; 2 BIOLAB RESEARCH srl, via E. Mattei 3, 28100 Novara, Italy; 3 Department of Neurology and ALS Centre, University of Piemonte Orientale, Maggiore della Carità Hospital, Corso Mazzini 18, 28100 Novara, Italy

**Keywords:** Amyotrophic lateral sclerosis, Neurodegeneration, Biomarker, Microbiota

## Abstract

**Background:**

A connection between amyotrophic lateral sclerosis (ALS) and altered gut microbiota composition has previously been reported in animal models. This work is the first prospective longitudinal study addressing the microbiota composition in ALS patients and the impact of a probiotic supplementation on the gut microbiota and disease progression.

**Methods:**

Fifty patients and 50 matched controls were enrolled. The microbial profile of stool samples from patients and controls was analyzed via PCR-Denaturing Gradient Gel Electrophoresis, and the main microbial groups quantified via qPCR. The whole microbiota was then analyzed via next generation sequencing after amplification of the V3–V4 region of 16S rDNA. Patients were then randomized to receive probiotic treatment or placebo and followed up for 6 months with ALSFRS-R, BMI, and FVC%.

**Results:**

The results demonstrate that the gut microbiota of ALS patients is characterized by some differences with respect to controls, regardless of the disability degree. Moreover, the gut microbiota composition changes during the course of the disease as demonstrated by the significant decrease in the number of observed operational taxonomic unit during the follow-up. Interestingly, an unbalance between potentially protective microbial groups, such as Bacteroidetes, and other with potential neurotoxic or pro-inflammatory activity, such as Cyanobacteria, has been shown. The 6-month probiotic treatment influenced the gut microbial composition; however, it did not bring the biodiversity of intestinal microbiota of patients closer to that of control subjects and no influence on the progression of the disease measured by ALSFRS-R was demonstrated.

**Conclusions:**

Our study poses the bases for larger clinical studies to characterize the microbiota changes as a novel ALS biomarker and to test new microbial strategy to ameliorate the health status of the gut.

**Trial registration:**

CE 107/14, approved by the Ethics Committee of the “Maggiore della Carità” University Hospital, Italy.

## Background

Amyotrophic lateral sclerosis (ALS) is a devastating, incurable neurodegenerative disease that affects the upper and the lower motor neurons leading to death by respiratory failure within 2–5 years from the onset of the disease. The etiology is still unknown, and the pathogenesis remains unclear. ALS is familial in the 10% of cases with a Mendelian pattern of inheritance while in the remaining sporadic cases a multifactorial origin is supposed in which several predisposing genes interact with environmental factors in manifesting the disease [1]*.* Imbalance in the gut microbiota composition may be one of the environmental factors contributing to the development of ALS. The composition of the intestinal microbiota is gaining importance in human health studies since there is increasing evidence that its alteration plays a role in disease etiology. The gut microbiota represents an important boundary between the environment and the immune system, and a major site for exposure to a wide range of both pathologic and intrinsic antigen and toxin production. It has been hypothesized that the intestinal microbiota can represent an epigenetic entity that interacts with environmental factors in determining pathogenic influence also on the central nervous system (CNS) [2].

Emerging evidences link alterations of the gut microbiota to the risk and the severity of some neurodegenerative diseases, such as in Parkinson’s disease (PD), where patients showed a lower abundance of Prevotellaceae members with respect to controls [3] and a correlation between specific taxa and different motor phenotypes [3, 4], and in Alzheimer’s disease (AD), finding differences in some microbial groups (e.g., Actinobacteria, Lachnospiraceae, *Rominococcus*, *Bacteroides*) compared to controls [5].

Several studies have hypothesized a role of the gut microbiota in the alteration of circulating levels of inflammatory cytokines or in the production of neurotoxins, which are known to affect the CNS and may have a role in the development or progress of neurological disorders [3, 6–9]*.* A correlation between ALS and an altered gut microbiota composition has previously been reported in animal models [10, 11], while only few preliminary studies have analyzed the composition of the fecal microbiota in ALS patients, with no conclusive results [12, 13]. Rowin et al. [13] showed, on a restricted number of patients, a lower Firmicutes/Bacteroidetes (F/B) ratio, used as a marker of intestinal dysbiosis, as well as a lower *Ruminococcus* spp. abundance in ALS patients with respect to controls. Brenner et al. [12], in 25 ALS patients, observed a higher OTU richness in ALS patients with respect to controls, without significant differences in biodiversity indices.

## Methods

This work is a prospective longitudinal study addressing the microbiota composition in ALS patients and matched controls with the aim to consider the possible impact of a probiotic supplementation on the gut microbiota and disease progression.

### Study design

This study was primarily designed as a prospective longitudinal study to evaluate the microbiota composition in a population of ALS patients compared with a case-control group of unrelated subjects matched for sex, age, origin, eating habits, and geographic region (if possible, unrelated members of the family, otherwise friends). The patients were then randomized, in a double-blinded, placebo-controlled, monocentric phase I pilot trial to receive a supplement or placebo in order to verify the changes of the microbiota composition with respect to the progression of the disease and the effects of the probiotic supplementation.

The study has been approved by the Ethics Committee of the “Maggiore della Carità” University Hospital (CE 107/14). All participants provided a written informed consent. The patients were enrolled at the tertiary ALS Centre in Novara, Italy, in a period from January 2016 to September 2017.

We enrolled 50 sporadic ALS patients with a diagnosis of probable or defined ALS according to El Escorial Criteria [14], aged 18 to 75 years, within 3 years from diagnosis, and force vital capacity percentage (FVC%) > 50%. We excluded patients with percutaneous endoscopic gastrostomy or nasogastric tube and with tracheotomy or non-invasive ventilation for more than 18 h/day, and who are unable to understand informed consent.

We also excluded patients with concomitant diseases (i.e., malignant neoplasms, gastrointestinal, inflammatory, autoimmune, cardiovascular, and respiratory disease) and subjects who have taken drugs or antibiotics that may modify the intestinal microbiota in the 8 weeks prior to recruitment. Patients received continuous riluzole treatment (100 mg/day) and symptomatic treatments. All patients were screened for the presence of mutations in the most common genes related to ALS (SOD1, C9Orf72, TARDP, FUS). All patients, at the baseline, underwent clinical and neurological evaluation that included the compilation of the ALS Functional Rating Scale–Revised (ALSFRS-R) score; spirometry with the measurement of FVC%; collection of body mass index (BMI) with a dietary assessment performed by an expert dietitian trained on ALS using an interview and a medical visit in order to collect the eating habits; the impairment of the autonomous feeding, chewing, and swallowing; and the weight loss compared to pre-morbid weight. Control subjects were matched for age and sex with the patients and were selected after a standardized interview by a trained researcher with the following characteristics: (1) living in the same geographic area; (2) same eating habits (food preferences specifically focused on the consumption of meat, vegetables, and sweets); (3) similar BMI; (4) no use of oral steroids, oral contraceptives, oral vitamin derivatives, antibiotics, probiotics, or herbal medicines that may affect the results during the last 4 weeks; (5) absence of concomitant diseases (e.g., malignant neoplasms, gastrointestinal, inflammatory, autoimmune, cardiovascular, and respiratory disease); and (6) voluntary participation in this clinical trial.

**Fig. 1:**
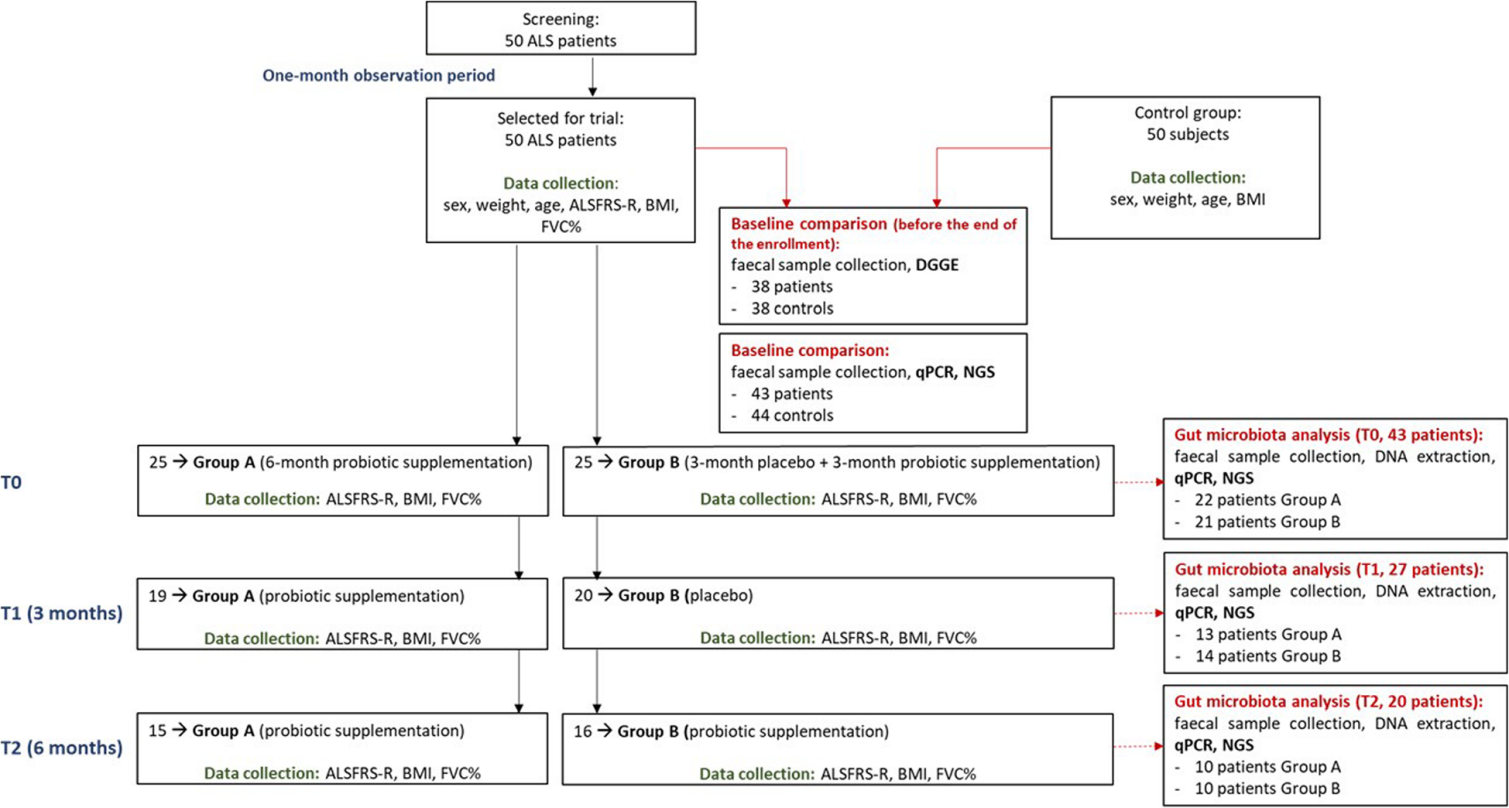
Study flowchart for baseline analysis and phase 1 trial. ALS, amyotrophic lateral sclerosis; T0, baseline; T1, 3 months from baseline; T2, 6 months from baseline; ALSFRS-R, ALS Functional Rating Scale–Revised; BMI, body mass index; FVC%, force vital capacity %; DGGE, denaturing gradient gel electrophoresis; qPCR, quantitative polymerase chain reaction; NGS, next-generation sequencing

After one observation month, patients were randomized to double-blind treatment either to the supplement or to placebo. The first group received a probiotic-based formulation for 6 months (group A), and the second one an equal dose of placebo for 3 months and then the probiotic-based formulation for the other 3 months (group B) (Fig. 1). Statistic unit assigned unique treatment code for all patients. Subjects, investigators, and clinical and laboratory staff were blinded to the treatment group assignment.

The follow-up considered monthly monitoring for 6 months. At each visit, the disease severity was assessed with the ALSFRS-R score, pulmonary function tests to calculate the FVC%, and a calculation of BMI performed by a dietitian. At each visit, dysphagia, eating habits, and caloric intake were also assessed.

Stool samples were collected at home from ALS patients at the baseline (T0), after 3 months (T1), and 6 months (T2) and from controls at the baseline. Sterile screw-top containers, usually used for stool culture for microbial detection, were used. In order to avoid contaminations, subjects did not touch the fecal material.

After DNA extraction from stool samples, DNA from patients and controls at T0 was analyzed via PCR-Denaturing Gradient Gel Electrophoresis (PCR-DGGE) analysis. Selected microbial groups from patients (T0, T1, T2) and controls (T0) were quantified via qPCR, and the whole microbial community were analyzed from the same samples via next generation sequencing (NGS) approach after amplification of the V3–V4 region of 16S rDNA.

### Probiotic supplement

The probiotic formulation is a mixture of five lactic acid bacteria administered in the following daily dosages: *Streptococcus thermophilus* ST10–DSM 25246, 5 × 10^9^ CFU/dose; *Lactobacillus fermentum* LF10–DSM 19187, 4 × 10^9^ CFU/dose; and *Lactobacillus delbrueckii* subsp. *delbrueckii* LDD01–DSM 22106, *Lactobacillus plantarum* LP01–LMG P-21021, and *Lactobacillus salivarius* LS03–DSM 22776, 2 × 10^9^ CFU/strain/dose*.* This probiotic formulation is ad hoc designed, patented, and produced by Probiotical SPA—Novara, Italy. The choice of the strains was done considering the results of previous studies that showed their capabilities to counteract gut pathogens, such as some *Candida* strains [15], Enterobacteria [16, 17], their anti-inflammatory properties and positive influence in restoring the gut physiological barrier [15, 18].

### DNA extraction from fecal samples

Stool samples were stored at − 80 °C until analysis. Total genomic DNA was extracted by using the QIAamp DNA Stool Mini Kit (Qiagen, West Sussex, UK) according to the manufacturer’s instruction with a slight modification of the standard protocol according to Aloisio et al. [19] and an additional treatment with lyticase (Sigma-Aldrich, Milan, Italy) at 37 °C for 30 min. The purity of extracted DNA was evaluated measuring the ratio of absorbance at 260 and 280 nm (Infinite®200 PRO NanoQuant, Mannedorf, Switzerland) and the DNA concentration estimated with the Qubit® 3.0 Fluorometer (Invitrogen, Life Technologies, Carlsbad, CA, USA).

### Absolute quantification of selected microbial groups using quantitative PCR

Absolute quantification of *Lactobacillus* spp., *Bifidobacterium* spp., *Clostridium* cluster I (including *C. baratii*, *C. hystoliticum*, *C. butyricum*, *C. prefringens*, *C. botulinum*, and *C. tetani*), *Escherichia coli*, Enterobacteriaceae, and total yeasts was performed with qPCR using the Fast SYBR®Green Master Mix (Applied Biosystems, Foster City, USA) and optimized concentrations of primers [20–23]. Standard curves were constructed using 16S rRNA PCR product of type strains of each target microorganism [23, 24], and data transformed to obtain the number of microorganism as Log CFU/g feces according to the rRNA copy number [25]*.* For total bacteria, the average of the 16S rRNA genes calculated on 10,996 records for bacteria according to rrnDB was used as the rRNA copy number [26, 27]*.*

For yeasts, considering that it is not possible to calculate the rRNA copy number, a normalization of the number of yeast cells was performed, before the conversion in Log CFU/g feces.

### PCR-DGGE

PCR-DGGE analysis was performed before the enrollment was completed on the first 38 control and 38 diseased subjects recruited in order to have a preliminary investigation of total Eubacteria and yeast populations. DNA was amplified using primers targeting the V2–V3 region of 16S rDNA and the D1 region of 26S rDNA, for Eubacteria and yeasts, respectively [28, 29]*.* DGGE analysis on obtained amplicons was performed as described previously [28, 30], using the Dcode System apparatus (Bio-Rad Laboratories, Hercules, CA, USA). Patterns were normalized by including a ladder with PCR products obtained from known pure cultures*.* Similarities and a cluster analysis among DGGE profiles were carried out using the Gel compare II v6.6 (Applied Maths, St. Martens-Latem, Belgium), by the unweighted pair-group method with the arithmetic average (UPGMA) clustering algorithm based on the Dice coefficient with an optimization coefficient of 1%.

### Preparation of DNA libraries for Illumina MiSeq sequencing

One hundred thirty-five DNA samples were subjected to Illumina sequencing. The V3–V4 region of the 16S rRNA gene was amplified and sequenced. One DNA sample belonging to group A and deriving from feces collected at T1 was excluded as it did not pass the established quality threshold. Forty-four samples belonged to the control group, 43 to the diseased group at T0, 13 to group A at T1, 14 to group B at T1, 10 to group A at T2, and 10 to group B at T2. The amplicons, approximately 460 bp in length, were generated using the forward and reverse primers, respectively: 5′-CCTACGGGNBGCASCAG-3′ and 5′-GACTACNVGGGTATCAATCC-3′ [31]. The assays were performed using a previously published protocol with some modifications [23]. The sequencing process was outsourced at Macrogen Inc. (Next Generation Sequencing Division), Seoul, Republic of Korea, using a 2 × 300 pair-end protocol.

### Bioinformatic and statistical analysis

Resulting 300 bp paired-end reads were assembled using FLASH [32]*.* Further sequence read processing was performed using QIIME ver. 1.9.1 [33] and ChimeraSlayer [34], including quality filtering based on a quality score of > 25 and removal of mismatched barcodes and sequences below length thresholds. Denoising, chimera detection, and clustering into operational taxonomic units (OTUs) (97% identity) were performed using USEARCH version 7 [34]*.* OTU sequences were aligned using PyNAST [35], and taxonomy assignment was determined using the SILVA SSU Ref database release 111 [36]*.* Biodiversity index analysis was performed using QIIME tools, in particular the script “core_diversity_analysis.py”; the phylogenetic classification of OTUs was carried out with the script “make_phylogeny.py” (fasttree). α-diversity was evaluated considering Chao, observed OTU, and PD whole tree metrics; β-diversity was evaluated using “weighted_unifrac” method [37]*.*

For phyla and families’ relative abundances and for α-diversity indices comparison, the normality and the homogeneity of variance of datasets were checked; statistical significance was evaluated with one-way ANOVA; two-way repeated measure ANOVA comparing different variance-covariance models was used to evaluate time-treatment interactions. Non-normal and non-homoscedastic datasets were compared with the Kruskall-Wallis test. For β-diversity indices comparison, data resulting from QIIME statistical elaboration were reported; the software performed 100 randomizations of sample/sequence assignments and recorded the probability that sample 1 is phylogenetically different from sample 2, using the UniFrac Monte Carlo significance test. The test was run for all pairs of samples. The *P* value was adjusted according to the Bonferroni correction taking into consideration the comparisons of interest within the study (*P* = 0.05/*n* of comparisons). In addition, principal coordinate analysis (PCoA) of the weighted UniFrac distance matrix was carried out.

F/B ratio values were calculated as indicator of dysbiosis, for each group of subjects [38]*.*

Canonical correspondence analysis (CCA), using the software CANOCO 4.5, was executed in order to detect interdependencies between the relative abundances of intestinal bacterial families and clinical and anthropometric data (ASLFRS-R score, FVC, BMI) considering longitudinal comparisons.

Data of microbial counts were subjected to Student’s *t* test to evidence significant differences between ALS patients and controls at baseline, and between treated and control group during the study period.

## Results

### Subjects

Between January 2016 and September 2017, 400 patients with ALS were screened at the tertiary ALS Centre in Novara, Italy. Fifty patients (28 males) and 50 matched controls (28 males) were enrolled. The mean age at entry was 60.24 (standard deviation (SD) 10.76) in patients and 53.60 (SD 15.34) in the control group. The mean weight was 67.09 (SD 13.28) with a BMI of 23.73 (SD 4.04) for ALS patients and 72.1 kg (SD: 13.67) with a BMI of 24.12 (SD 4.35) for controls.

After 1-month observation period, ALS patients were randomly assigned to receive either placebo (*n* = 25) or probiotics (*n* = 25). The demographic and clinical profiles of the two groups at entry are shown in Table 1 and were comparable (Table 1). The clinical features have remained comparable between group A and group B also at T1 and T2.

**Table 1:** Baseline clinical features of ALS patients for group A and group B

Clinical features	All patients (50)–T0		Analyzed patients (43)–T0		All patients (39)–T1		Analyzed patients (27)–T1		All patients (31)–T2		Analyzed patients (20)–T2	
	Group A	Group B	Group A	Group B	Group A	Group B	Group A	Group B	Group A	Group B	Group A	Group B
Number (sex, male)	25 (M 15)	22 (M 14)	22 (M 12)	21 (M 12)	19 (M 10)	20 (M 12)	13 (M 7)	14 (M 8)	15 (M 9)	16 (M 10)	10 (M 7)	10 (M 6)
Age (SD)	60.36 (10.86)	59.64 (8.12)	59.64 (8.12)	59.71 (10.03)	62.0 (11.15)	57.95 (11.25)	59.77 (10.05)	60.71 (10.09)	61.2 (11.47)	57.56 (11.58)	58.9 (10.88)	62.4 (11.80)
BMI (SD)	24.82 (3.95)	24.84 (3.97)	24.84 (3.97)	22.84 (4.01)	24.70 (4.03)	23.28 (4.18)	25.65 (4.39)	23.19 (4.44)	24.21 (3.86)	23.09 (3.96)	24.64 (4.19)	23.17 (4.53)
Spinal onset (*n*, %)	20 (80%)	18 (82%)	18 (82%)	17 (81%)	16 (84%)	17 (85%)	12 (92%)	12 (86%)	12 (80%)	14 (88%)	9 (90%)	10 (100%)
Disease duration (SD)	20.91 (10.89)	19.85 (10.71)	21.54 (10.90)	19.14 (10.70)	22.47 (9.70)	23.65 (9.98)	24.23 (9.16)	22.71 (9.60)	27.07 (9.65)	27.56 (10.14)	29.9 (9.26)	25.50 (9.28)
FVC% (SD)	81.48 (18.28)	83.90 (18.46)	83.90 (18.46)	85.52 (18.79)	72.26 (22.0)	74.22 (21.35)	80.0 (19.27)	78.67 (17.78)	64.71 (24.9)	74.57 (23.99)	68.67 (22.38)	81.0 (22.39)
ALSFRS-R (SD)	36.56 (5.82)	36.77 (5.88)	36.77 (5.88)	36.05 (5.86)	36.26 (7.55)	31.77 (7.26)	36.85 (7.55)	31.28 (7.02)	33.64 (9.24)	31.0 (9.10)	33.22 (7.99)	31.11 (8.33)
Bulbar ALSFRS-R (SD)	11.26 (1.53)	11.35 (1.52)	10.47 (1.49)	10.29 (1.52)	11.2 (1.73)	11.36 (1.23)	10.33 (1.54)	10.15 (1.72)	9.6 (2.42)	10.43 (1.98)	9.92 (2.36)	9.87 (1.97)
Delta ALSFRS-R (SD)	–	–	–	–	0.63 (1.36)	1.08 (1.43)	0.77 (1.38)	1.45 (1.55)	0.93 (1.05)	0.8 (0.99)	1.0 (0.88)	0.63 (0.77)

The values are expressed as mean (SD). *BMI* body mass index, *FVC* forced vital capacity, *ALSFRS-R* ALS Functional Rating Scale–Revised. *P* value: not statistically significant for each analysis (> 0.05). T0: baseline, T1: 3 months since treatment start, T2: 6 months since treatment start. The value of disease duration is in months. Delta ALSFRS-R is calculated as monthly progression

Two patients enrolled in the study and without a family history for ALS or frontotemporal dementia showed the GGGGCC hexanucleotide expansion in the first intron of *C9orf72*.

Six samples from controls and 7 from patients at T0 had to be excluded from the analyses because extracted DNA did not have the necessary quality to be amplified for NGS, probably due to improper conservation of fecal samples prior to their delivery to the clinic.

One patient died for the progression of the disease. Twenty patients discontinued the study before the conclusion of their 6 months follow-up period and were documented as dropouts.

### Baseline characteristics

#### PCR-DGGE

**Fig. 2:**
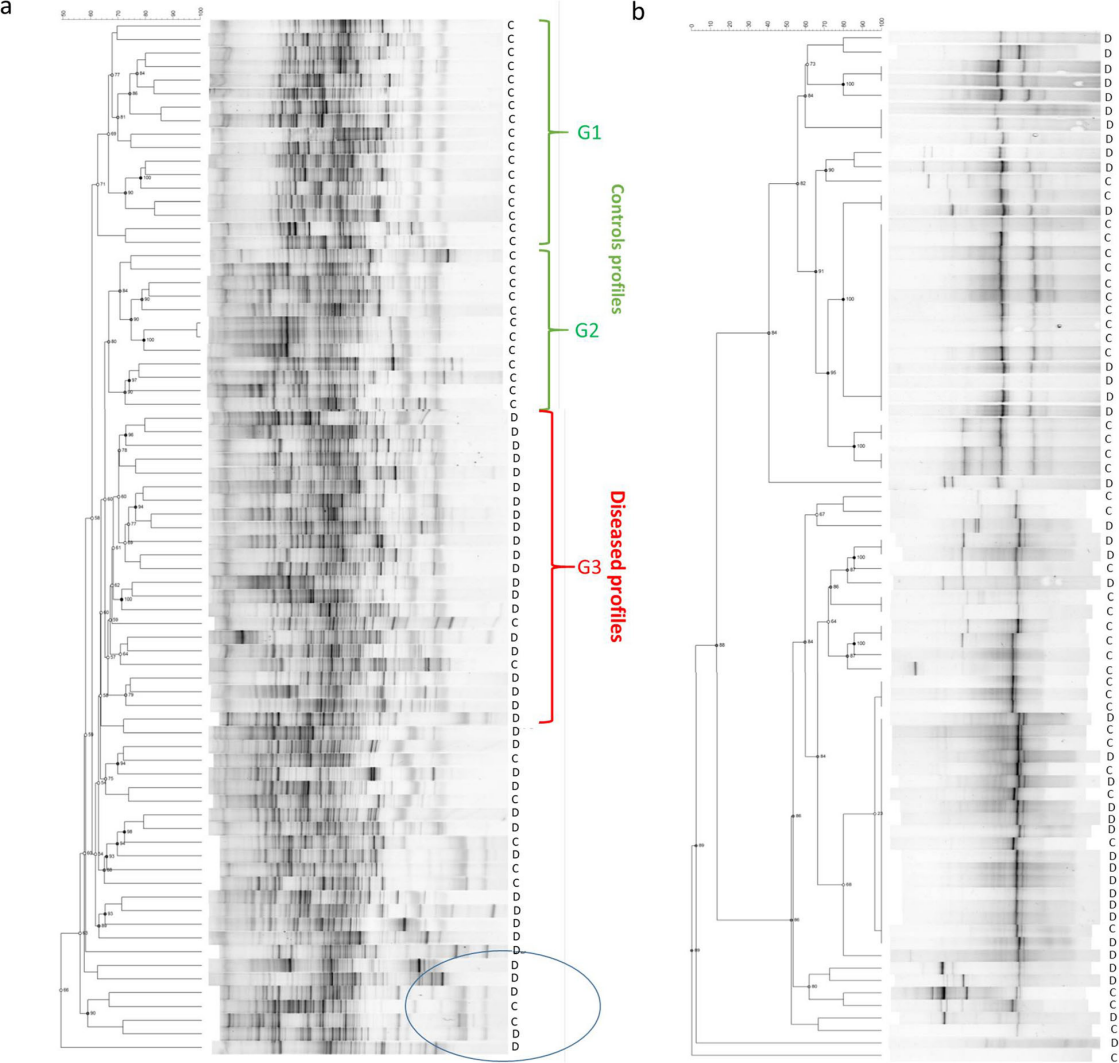
Gut bacteria and yeast profiles at baseline obtained by DGGE. **a** UPGMA dendrogram and DGGE profiles of Eubacteria. Three main cluster groups are indicated as G1, G2, and G3: G1 and G2 represent controls (C) and G3 represents diseased subjects (D). **b** UPGMA dendrogram and DGGE profiles of total yeasts in controls (C) and diseased (D) subjects

The cluster analysis of the 16S rDNA generated by DGGE using the UPGMA algorithm is shown in Fig. 2a. The fingerprint of the intestinal Eubacteria was characterized for each subject by 30–40 detectable bands, which differed in number, position, and intensity. Except for 7 profiles (5 diseased and 2 controls) forming a unique group with similarity lower than 59% (bottom part of Fig. 2a), two major clusters were obtained: one grouping 17 control subjects (similarity ~ 60.6%, group G1 in Fig. 2a) and one larger containing 3 sub-clusters—the first one (group G2) composed of 12 control subjects; the second one including 20 diseased samples (group G3), i.e., more than 50% of the total diseased patients considered in the analysis, and 2 control profiles (similarity less than 65.2%); and the third one containing 14 diseased samples and 5 control ones.

Yeast profiles, obtained from the cluster analysis of the amplified D1 region of 26S rDNA, are much simpler than those of Eubacteria (Fig. 2b). Overall, no unequivocal grouping of diseased/control profiles could be observed. The dendrogram obtained from the DGGE banding patterns showed two distinct sub-clusters, according to the number of bands as well as their positions, each composed of both control and diseased subjects.

#### qPCR

DNA quantification after extraction from the same amount of stool showed, at the baseline, a lower DNA concentration in patients compared with controls (Table 2). However, the number of total bacteria was not significantly different in the two groups. A significant lower amount of *Clostridium* cluster I and yeasts and a significantly higher concentration of *E. coli* were detected in ALS patients with respect to controls; Enterobacteriaceae were higher in ALS subjects (*P* = 0.05). In addition, higher values of FVC% and ALSFRS-R significantly correlated with a greater amount of yeasts in the microbiota (*P* < 0.001 and 0.03, respectively). No significant correlations were found with the BMI.

**Table 2:** DNA concentration and mean counts of the analyzed microbial groups by qPCR in stool samples of ALS patients (case) and controls

	Sample	Mean (SD)	*P* value
DNA	Case	155.50 (118.68)	**0.02**
	Control	210.10 (97.25)	
Total bacteria	Case	10.36 (0.86)	0.90
	Control	10.34 (0.74)	
*Lactobacillus* spp.	Case	5.44 (1.26)	0.09
	Control	5.77 (0.76)	
*Bifidobacterium* spp.	Case	7.30 (1.63)	0.54
	Control	7.43 (1.28)	
*E. coli*	Case	6.60 (1.13)	**0.04**
	Control	6.0 (1.61)	
*Clostridium* cluster I	Case	5.72 (1.55)	**0.01**
	Control	6.39 (0.84)	
Enterobacteriaceae	Case	8.51 (0.8)	**0.05**
	Control	7.96 (1.84)	
Total yeast	Case	5.78 (0.81)	**0.02**
	Control	6.07 (0.65)	

The DNA concentration is expressed as ng/200 mg of feces, and the mean counts as Log CFU/g of feces; the related *P* value is reported. Bolded values indicate *P* ≤ 0.05

#### NGS analysis

**Fig. 3:**
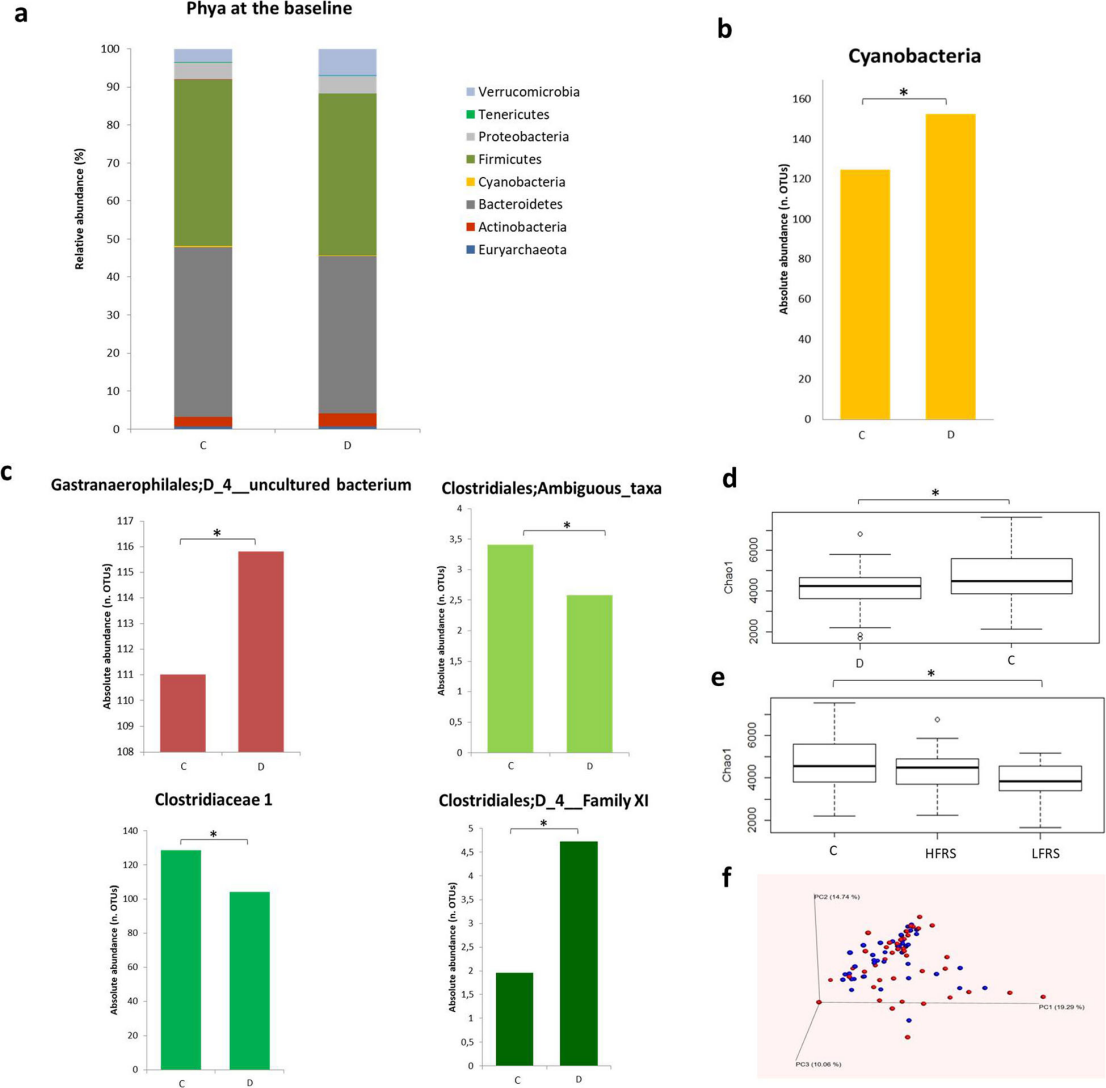
Gut bacterial characteristics at the baseline and main differences between controls and diseased subjects. **a** Relative abundance of the main bacterial phyla in the control group. **b** Relative abundance of the main bacterial phyla in diseased group. **c** Differences (*P* < 0.05) in Cyanobacteria between controls (C) and diseased (D) subjects; data are expressed as absolute abundance (number of OTUs). **d** Bacterial groups classified at family level showing significant differences (*P* < 0.05) between controls (C) and diseased (D) subjects. **e** Differences (*P* < 0.05) in α-diversity Chao1 index between diseased (D) and controls (C). **f** Differences (*P* < 0.05) in α-diversity Chao1 index in ALS patients subgrouped for their ALSFRS-R scale values (HFRS ≥ 35, LFRS < 35). **g** PCoA representing β-diversity among individuals at baseline (controls = red, ALS subjects = blue)

A total dataset of 13,592,139 filtered high-quality joined reads was generated after sequencing the V3–V4 region of 134 DNA samples, obtaining about 101,433 sequences per sample. Only 0.46% of sequences had a mean sequence quality (Phred score) under 25. The rarefaction depth was 49,795. Figure 3 a and b show the major phyla belonging to Bacteria and Archaea in control and diseased subjects. The more representative phyla were Bacteroidetes and Firmicutes in both groups, which showed a relative abundance of 40–45%. A higher percentage of Actinobacteria and Verrucomicrobia was detected in the diseased group (3.5 and 6.6%, respectively) compared to the control one (2.6 and 3.6%, respectively), although the difference was not statistically significant (*P* = 0.94 and *P* = 0.25 respectively). Members of the Cyanobacteria phylum were significantly higher (*P* < 0.05) in the diseased group with respect to the control one (0.3% vs 0.2%, respectively) (Fig. 3c, Additional file 1: Table S1). The distribution of raw OTUs of the most abundant phyla among controls and diseased subjects is reported in Additional file 1: Figure S1.

In addition, the heat map presented in Additional file 1: Figure S3a shows several differences at the family level between the two groups. Some families presented a higher relative abundance in the diseased group, even though not of statistical significance: Clostridiales vadinBB60 group belonging to Clostridia, Bacteroidales S24-7 group, Coriobacteriaceae, Verrucomicrobioaceae, and Lactobacillaceae. On the other hand, the control group showed a higher relative abundance of Veillonellaceae, Promicromonosporaceae, and Peptostreptococcaceae.

The difference in the Cyanobacteria phylum is reflected at the family level in the significantly higher abundance in ALS patients of Gastranaerophilales (uncultured bacteria) (*P* < 0.05) (Fig. 3d, Additional file 1: Table S1). A significantly different amount was also found in families related to Clostridiaceae, such as Clostridiales Ambiguous taxa and Clostridiaceae 1, which were lower in patients (*P* < 0.05) whereas Clostridiales Family XI resulted higher in patients (*P* < 0.05) (Fig. 3d, Additional file 1: Table S1). The distribution of raw OTUs of the significant families among controls and diseased subjects is reported in Additional file 1: Figure S2.

At the genus level (Additional file 1: Figure S3b), *Lactobacillus*, *Citrobacter*, *Coprococcus*, and some genera belonging to Ruminococcaceae (including *Ruminiclostridium*) were found to be more abundant in patients. In addition, genera belonging to Enterobacteriaceae (such as *Escherichia* and *Shigella)*, *Akkermansia*, *Eubacterium eligens* group, *Odoribacter*, *Bifidobacterium*, *Pseudoflavonifractor*, and other genera belonging to Prevotellaceae and Ruminococcaceae, specifically Ruminococcaceae NK4A214 group and Ruminococcaceae UCG-014, manually annotated as *Intestinimonas* and members of the family Hungateiclostridiaceae, respectively, were also more abundant in ALS patients. Genera belonging to Veillonellaceae and Lachnospiraceae (Lachnospiraceae_Eubacterium) families, the genus *Parasutterella*, *Ruminococcus* and *Subdogranulum*, both belonging to Ruminococacceae, were, on the contrary, more abundant in the control group. Two genera belonging to Gastranaerophilales were more abundant in diseased subjects (*P* < 0.05); all the other differences at the genus level were not statistically significant (*P* = 0.05 for *Ruminiclostridium*).

Differences in the microbial community biodiversity between controls and diseased subjects were observed by calculating the value of α- and β-diversity. The Chao1 index (α-diversity), related to the abundance of sequences for each OTU, was significantly higher (*P* < 0.05) in the control group with respect to diseased subjects (Fig. 3e). The other α-diversity indices, observed OTU and PD whole tree, were not significantly different between the two groups. Significant differences (*P* < 0.05) in the Chao1 index were also detected subgrouping ALS patients for their ALSFRS-R scale. Patients were classified in High Functional Rating Scale (HFRS) with score ≥ 35 and Low Functional Rating Scale (LFRS) < 35. We considered 35/48 as moderate level of disability (> 35/48: at least partial autonomy, < 35/48: at least partial dependent by caregiver). The statistical analysis showed significant differences between the control group and LFRS patients (Fig. 3f), whereas no differences were found between HFRS and LFRS patients (*P* > 0.05). β-diversity was also significantly different (*P* < 0.05) between controls and ALS patients, even if the PCoA analysis did not show a clear division between the two groups, but a scattered trend for each individual, especially for controls (Fig. 3g). Statistically significant differences in β-diversity resulted between the control and HFRS group (*P* < 0.005). Moreover, within the diseased group at the baseline, differences were observed between HFRS and LFRS (*P* < 0.05), particularly *Eubacterium eligens* group was more abundant in HFRS with respect to LFRS. Patients with C9Orf72 expansion did not differ in all examined parameters.

### Probiotic/placebo supplementation in ALS subjects

#### qPCR

No adverse events (AEs) attributed to probiotic supplementation and no AEs of special concern, such as diarrhea or gastrointestinal symptoms, occurred. No patient modified the diet in terms of contents and macronutrients during the follow-up.

The same microbial groups analyzed at the baseline (T0) were quantified at T1 (3 months) and T2 (6 months), (Additional file 1: Table S3). No significant differences were observed in total bacteria counts, although at T1 lower bacterial counts were observed in group B with respect to group A. DNA concentration extracted from 200 mg of fecal material decreased, although not significantly, with the progression of the disease. A significant reduction of yeast concentration in T2 group A with respect to T2 group B (*P* = 0.03, Additional file 1: Table S3 and Figure S4) was found. No significant differences were observed for the single bacterial groups, except for an increase (*P* = 0.05) of *E. coli* in group B patients with respect to the group of patients (group A) that received the probiotic for 6 months. No significance was also observed by correcting the data for the time-treatment interaction. Observed changes in ALSFRS-R, FVC%, and BMI did not differ between the two groups.

#### NGS analysis

**Fig. 4:**
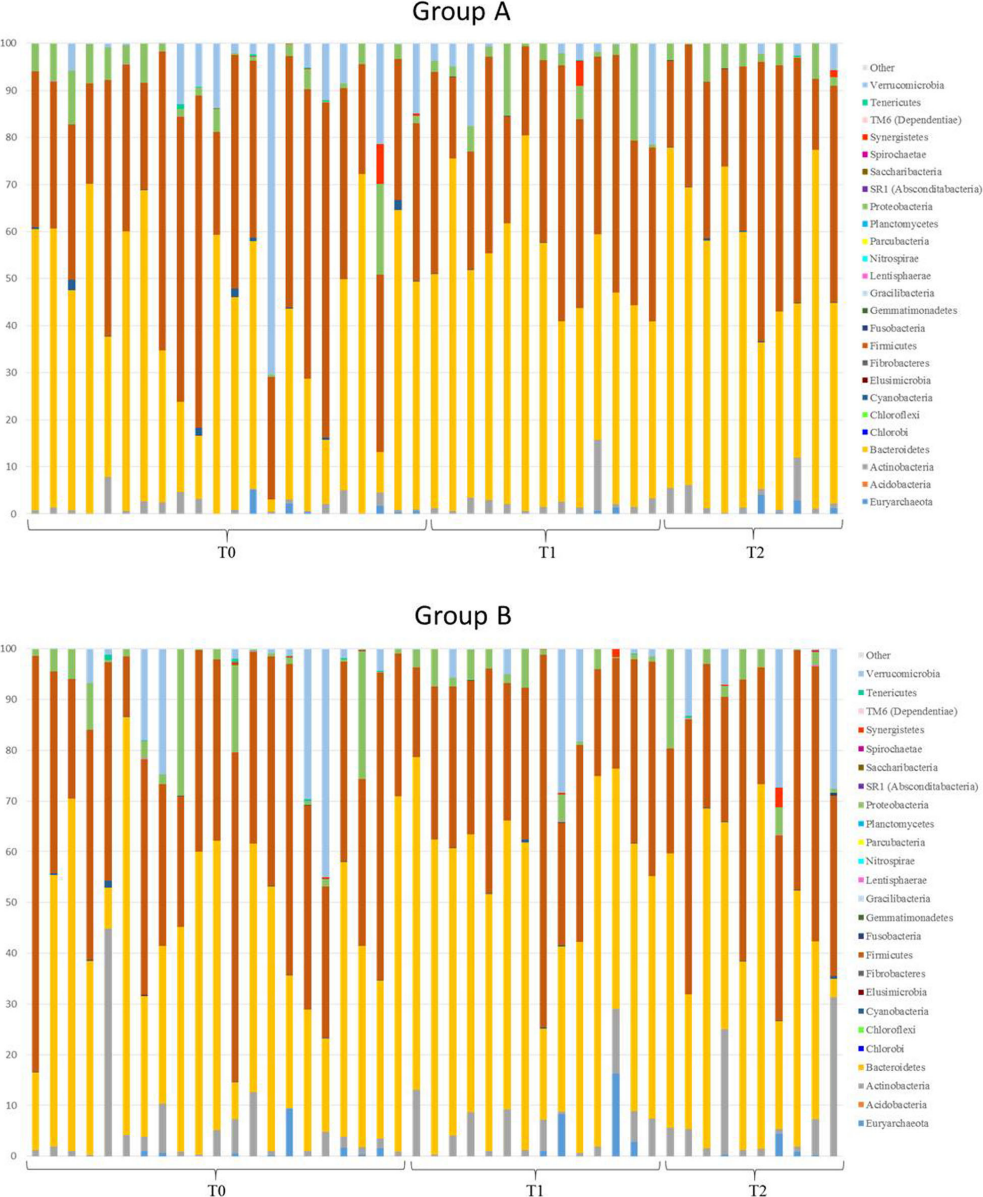
Relative abundance of gut microbial phyla in ALS patients during the intervention. Group A consists of subjects receiving the probiotic treatment for 6 months (T0 = baseline, T1 = 3 months from baseline, T2 = 6 months from baseline). Group B consists of subjects receiving the placebo treatment for 3 months (T1) and the probiotic treatment for the following 3 months (T2). Phyla with a percentage of relative abundance less than 0.002% are grouped in “other”

Figure 4 shows the relative abundance at the phylum level of each ALS patient before and after each treatment at the different times. A large variability among individuals was detected. The abundance of Cyanobacteria decreased over time in both the probiotic and placebo groups, although not significantly. Euryarchaeota and Actinobacteria were more represented in the group of patients that received the probiotic formulation after an initial treatment with placebo (T2 group B) with respect to the other groups. Moreover, some individuals presented higher levels of Synergistetes members (2–5%) with respect to the baseline independently of the treatment.

**Fig. 5:**
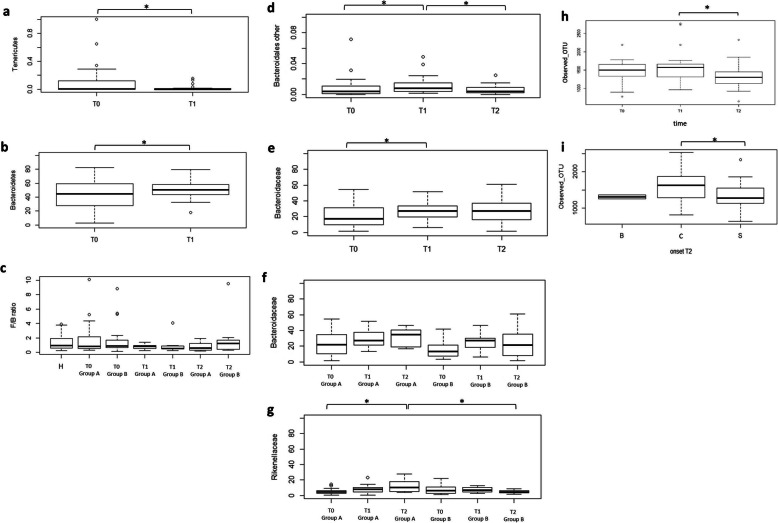
Gut bacterial characteristics and main differences within the study groups during the intervention. **a** Differences (*P* < 0.05) in Tenericutes relative abundance after 3 months (T1), not considering the type of treatment. **b** Differences (*P* < 0.05) in Bacteroidetes relative abundance after 3 months (T1), not considering the type of treatment. **c** F/B ratio in the different time-treatment groups. **d** Differences (*P* < 0.05) in relative abundance of other families belonging to Bacteroidales within time, not considering the type of treatment. **e** Differences (*P* < 0.05) in Bacteroidaceae relative abundance within time, not considering the type of treatment. **f** Relative abundance of Bacteroidaceae in ALS patients considering the time and the type of treatment. **g** Differences (*P* = 0.05) in Rikenellaceae relative abundance in patients considering the time and the type of treatment. **h** Differences in α-diversity index observed OTU (*P* < 0.05) within time, not considering the type of treatment. **i** Differences in α-diversity index observed OTU (*P* < 0.05) considering the controls (C) and patients at T2 subgrouped in spinal (S) and bulbar (B) onset

At the phylum level, considering time as the only variable not including the different type of treatment, the analysis showed a significant decrease of one of the less abundant phyla, Tenericutes, and a significant increase of one of the major phyla, Bacteroidetes, at T1 with respect to T0 (Fig. 5a, b, Additional file 1: Table S2). All groups of individuals were compared considering the Firmicutes/Bacteroidetes ratio (Fig. 5c). No statistically significant differences were obtained in the F/B values, although the value was the highest in T2 group B.

At the family level, the most represented families were Bacteroidaceae, followed by Ruminococcaceae, Lachnospiraceae, and Rikenellaceae (Additional file 1: Table S4). Bacteroidaceae, together with other families belonging to Bacteroidales, significantly changed over time, not considering the type of treatment (Fig. 5e, d, Additional file 1: Table S2). Bifidobacteriaceae transitorily increased after 3 months of probiotic treatment in both ALS patient groups, although not significantly. Bacteroidaceae increased in all patients, but particularly in group A (Fig. 5f). A significant increase in Rikenellaceae relative abundance was associated to 6-month probiotic treatment (Fig. 5g); moreover, group A T2 had considerable higher levels of Rikenellaceae (12.25%) with respect to group B T2 (4.82%) (Additional file 1: Table S2 and S4). The distribution of raw OTUs of the significant families among diseased subjects is reported in Additional file 1: Figure S5.

A relevant drop of Prevotellaceae, Christensenellaceae, and Clostridiales vadinBB60 group was detected in group A T2. The family of Clostridiaceae 1 decreased in both groups after 6 months from baseline, thus increasing the difference from the value associated to controls (Additional file 1: Table S4). Lachnospiraceae only diminished in group A. Ruminococcaceae showed the same trend in the two ALS groups, decreasing at T1 and then increasing at T2. Regarding Veillonellaceae, a stronger reduction was detected in group B at T1; in any case, the abundance remained higher in controls. Interestingly, the Verrucomicrobiaceae levels resulted higher in ALS patients.

Regarding α-diversity indices, a significant decrease was reported in the number of observed OTU in ALS patients between 3 (T1) and 6 months (T2) from baseline, not considering the type of supplementation but only time as a variable (Fig. 5h). The number of observed OTU was also significantly lower in ALS subjects with a spinal onset at 6 months from baseline (T2), compared to controls (Fig. 5i). No differences were observed for Chao1 and PD whole tree indices. The β-diversity analysis showed significant differences in group A at T1 and T2 with respect to the baseline. Patients subjected to 3 months probiotic treatment (T2 group B) showed significant differences compared to the previous placebo treatment (T1 group B). Moreover, differences were observed between the group receiving the probiotic for 3 months (T2 group B) and the group receiving the probiotic for 6 months (T2 group A) (Table 3). The β-diversity was not significantly different subgrouping patients for ALSFRS-R score; however, the control group was significantly different both from the HFRS and LFRS groups at the baseline (*P* < 0.05).

**Table 3:** β-diversity comparisons between different groups of subjects

Group 1	Group 2	*P* value	*P* value_corr_
T0 all cases	Control	0.001	**0.001**
T0 all cases	T1 group A	0.001	**0.014**
T0 all cases	T1 group B	0.008	0.112
T0 all cases	T2 group A	0.001	**0.014**
T0 all cases	T2 group B	0.024	0.336
T1 group A	T2 group A	0.005	**0.070**
T1 group B	T2 group B	0.002	**0.028**
T1 group A	T2 group B	0.001	**0.014**
T1 group B	T1 group A	0.047	0.658
T1 group B	T2 group A	0.001	**0.014**
T2 group B	T2 group A	0.001	**0.014**

“*P* values_corr_” are the adjusted *P* values calculated with the Bonferroni correction for the comparisons considered. Bolded values indicate statistical significance

**Fig. 6:**
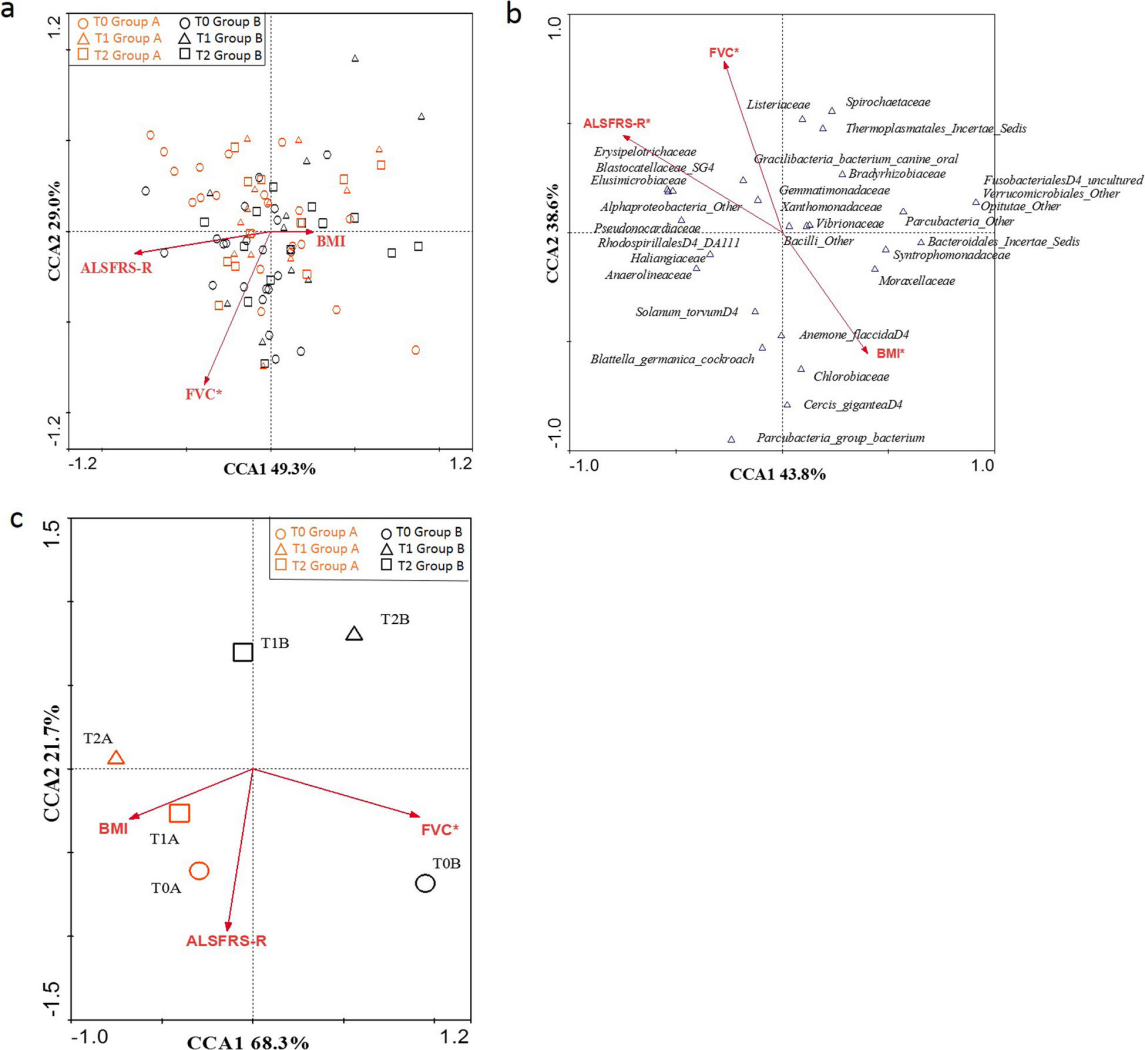
CCA on multivariate association between gut microbial families and ALSFRS-R, BMI, and FVC parameters. **a** Plot of all patients during the study. **b** Plot representing the bacterial families that influenced the most the parameters considered (ALSFRS-R, BMI, and FVC). **c** Plot representing the trend of study groups

The multivariate CCA on cases, using as variables ALSFRS-R scores, FVC, and BMI measures collected during the follow-up (T0, T1, T2) and as dataset the relative abundance of families, was performed (Fig. 6a). Only FVC was significantly related to the microbiota composition of patients regardless of treatment and time (*P* = 0.034, *R* = − 0.845 AX2), although also ALSFRS-R score seemed to be strongly related to the variability expressed by CCA1 (*R* = − 0.809 AX1). After identifying with the CANOCO software the families that influenced the most the trend of the variables considered, the analysis was repeated using in the dataset only the selected families (Fig. 6b). All the clinical parameters analyzed resulted significant in influencing the microbiota composition, in particular for what concerns taxa with low relative abundances.

Considering longitudinal comparisons (T0 group A, T0 group B, T1 group A, T1 group B, T2 group A, T2 group B) on the dataset constructed on families, interesting divisions among the study groups were observed (Fig. 6c). In relation to FVC, which remained statistically significant (*P* = 0.002, *R* = 0.919 AX1), a division between patients belonging to group B (T1B, T2B) and patients belonging to group A (T1A, T2A) was observed, suggesting a different respiratory function in these two macro-groups. In addition, a further separation of the two baseline groups (T0A and T0B) from the others was described. Moreover, considering the ALSFRS-R score, a division between group A and group B at different timepoints was observed, although not statistically significant. No correlation was found with the BMI.

## Discussion

Pre-clinical studies demonstrated that gut microbiota composition is altered in murine ALS model [10, 11] and an association between repeated antibiotics use and an increased risk of ALS has been reported as well [39]. To the best of our knowledge, only two studies have focused on the intestinal microbiota of ALS patients [12, 13], collecting information on controls and diseased subjects without reaching a definitive conclusion on gut dysbiosis in ALS and not considering possible variations of the microbiota with disease progression. The power of our study relies on (1) the consistent number of subjects enrolled according to a case-control method (fifty phenotypically and genetically well-characterized ALS patients and 50 matched controls were enrolled; 43 diseased and 44 control samples were examined), (2) the consideration of possible modifications of the microbiota composition during the course of the disease performing multiple analysis during a 6-month period, (3) the supplementation for the first time of a probiotic formulation to ALS patients, and (4) the microbiota analysis performed through an integrated molecular approach that includes PCR-DGGE, NGS, and qPCR.

### Baseline

The significantly lower DNA amount in ALS patients with respect to controls is not due to a reduced bacterial count but can be ascribed, at least partially, to the count of total yeasts that was significantly higher in controls. However, it cannot be excluded that epithelial cell DNA may influence the amount of total DNA, due to a higher epithelial cell turnover in controls with respect to ALS patients [40]*.*

DGGE bacterial profiles showed a great variability both in controls and diseased subjects. Their comparison does not show a clear shift in the intestinal microbiota composition in the diseased patients with respect to the controls. This is also in agreement with the not significant alteration of the F/B ratio, considered by some authors as an indicator of fecal microbiome composition [12]*.* However, the DICE-UPGMA analysis of DGGE profiles, mainly based on the presence/absence of bands, showed a well-defined cluster division, except for some samples, between diseased and controls. This analysis allowed us to conclude that differences in the predominant bacterial composition between controls and diseased individuals do exist as also confirmed by the reduced α-diversity calculated with Chao1 index. The decrease in OTU abundance was particularly evident comparing controls and patients with a greater disability (low ALSFRS-R score). Although Chao1 is only one aspect of intra-individual diversity, its decrease may indicate a selection of certain microbial groups with the disease progression. Furthermore, the groups of controls and ALS patients, regardless of clinical severity, resulted significantly different considering inter-individual diversity (β-diversity), as shown by the PCoA analysis. On the contrary, the DGGE yeast profiles did not show appreciable qualitative differences, although lower yeast levels were detected in patients than in controls. Interestingly, the yeast amount was higher in patients with a lower degree of disability, thus confirming the positive role of yeasts as gut beneficial commensals [41]*.*

From the NGS analysis, Bacteroidetes and Firmicutes were the most abundant phyla, as expected [42], in both controls and diseased subjects. A very interesting result of our study is the demonstration that Cyanobacteria, at phylum level, were significantly more abundant in ALS patients compared to controls. This trend is also reflected at the family and genus level. These data support the hypothesis that Cyanobacteria play a fundamental role in the pathogenesis of neurodegenerative diseases and particularly of ALS. The Cyanobacteria hypothesis emerged from studies carried out in Guam, which concluded that the non-protein amino acid β-methylamino-l-alanine (BMAA), derived from a tropical plant, is likely responsible for a disease complex consisting of a sporadic form of ALS combined with PD and dementia (ALS/PDC) [43–48]. BMAA was found to originate from symbiotic Cyanobacteria in the roots of *Cycas micronesica*, and it biomagnified in the food chain through animals (flying foxes, pigs, deers) up to man. Murch et al. demonstrated that BMAA is higher in human brains of patients with ALS/PDC with respect to controls [48]*.* The mechanism of action of BMAA may be linked to the glutamate hypothesis of ALS, for which the exposure to excitatory amino acids (glutamate and aspartate) stimulates glutamate receptors, resulting in excessive intracellular calcium ion accumulation and consequently motor neuron death [49–53]. Furthermore, Cyanobacteria are responsible for the production of other neurotoxic molecules, such as saxitoxin that can lead to paralysis of voluntary musculature [54, 55], microcystins that are toxic for brain [56, 57], and nodularin that cause cytoskeletal damage [58]*.* Our results can constitute a starting point for an investigation of cytotoxic-related Cyanobacteria metabolites in blood of ALS patients to support the hypothesis of the involvement of these bacteria in the pathogenesis of ALS. On the contrary, the genera *Lactobacillus*, *Bifidobacterium*, and *Odoribacter*, all known to metabolize glutamate, were more abundant in ALS patients, as previously reported also by Rowin et al. [13]*.* These findings allowed us to speculate that these bacteria may be involved in the removal of potential neurotoxic substances and pose theoretical basis for the use of a probiotic supplementation as a complementary therapeutic strategy.

Another important result of our study is the demonstration of the imbalance of some intestinal bacteria that play important roles in the immunomodulation of the CNS. The role of innate and adaptive immune response and inflammation in the pathogenesis of ALS is well known. Verrucomicrobia phylum, Verrucomicrobiaceae family, and the *Akkermansia* genus, belonging to this family, are higher in ALS patients, as previously reported also in multiple sclerosis patients [9]*. Akkermansia* has been correlated to pro-inflammatory pathways including upregulation of genes involved in antigen presentation, B and T cell receptor signaling, and activation of complement and coagulation cascades [59]*.* Hence, a high amount of this microbial group may contribute to the inflammatory condition in ALS. Moreover, we found lower levels of Veillonellaceae in ALS patients than in controls as previously reported in multiple sclerosis [7]*.* Veillonellaceae are phylogenetically related to *Clostridium*, which induces regulatory T cells [60]*.* Therefore, the lower abundance of Veilloneaceae in patients can be linked to a compromised maintenance of immune homeostasis. The higher abundance of some genera belonging to Enterobacteriaceae, such as *Citrobacter* and *Escherichia-Shigella*, in our patients may contribute to intestinal inflammation [61, 62]*.* The increase in fecal Enterobacteriaceae has been also reported in patients with major depressive disorders, accompanied by a low level of brain-derived neurotrophic factor (BDNF) in the blood [63, 64]*.*

Different trends were shown for some families belonging to Clostridiales. Within Clostridiaceae, the significantly lower amount of Clostridiaceae 1, including some ambiguous taxa belonging to this family, as well as *Clostridium* cluster I in ALS patients is in agreement with the results previously reported by Rowin et al. [13]*.* Differently, the Peptostreptococcaceae family (synonymous of Clostridium Family XI) were overrepresented in patients as also observed in the guts of colorectal cancer patients [65]*.* Shifts in the Clostriales profile have been also reported in children with neurodevelopmental dysfunctions [66, 67]*.*

Members of Eubacteriaceae, in particular the *Eubacterium eligens* group, and of Ruminococcaceae, principally *Ruminococcus* and *Subdoligranulum* genera, which are involved in the degradation of plant cellulose and hemicellulose in the host [68], were found to be lower in our ALS patients than in controls Their depletion can be associated to a reduced production of short chain fatty acids (SCFAs) and, consequently, to a lower energy provision in the host*.* The *Eubacterium eligens* group, which we found more abundant in ALS patients, is able to produce equol, an estrogen derived from the metabolism of dietary daidzein [69], known to have a neuroprotective activity [70]*.* Therefore, these bacteria may have a protective role against the progression of the disease, as shown by their higher relative abundance in patients with lower disability.

The higher levels of *Citrobacter* in patients may also be involved in the pathogenesis of ALS. This genus, in particular *Citrobacter rodentium*, has been demonstrated to exert a pathogenic mechanism similar to enteropathogenic and enterohemorrhagic *E. coli* [71]. Moreover, *C. rodentium* infection in mice induces colitis and dysbiosis characterized by an overgrowth of *C. rodentium* and a reduction in the abundance and overall diversity of the resident microbiota [72]*.* Members of the *Pseudoflavonifractor* genus, more abundant in ALS patients, possess an anorectic function [73]*.* Therefore, this data can be linked to a losing weight trend associated to ALS [74].

### Follow-up and probiotic intervention

Previous studies [75] have suggested that probiotic bacteria, besides restoring a possible microbial imbalance, can be considered as delivery vehicles for neuroactive compounds representing a possible therapeutic/preventive strategy in neurological diseases. Our results showed no substantial alterations in the gut microbial composition associated to the administered probiotic treatment, and no significant alterations in the fecal microbiota composition indicators were observed. Only the Rikenellaceae family, one of the most represented microbial family in the gut and belonging to Bacteroidales, significantly increased with the 6-month probiotic treatment (Fig. 5g); as its relative abundance was significantly higher at T2 group A with respect to T2 group B, we can conclude that the duration of the probiotic treatment may influence the gut microbial composition. Moreover, since Rikenellaceae members are involved in propionate production [76], this probiotic administration may be able to affect SCFA production. The Bacteroidaceae increase, although not significant, in both groups, but noticeably in group A (Fig. 5f), may confirm that the longer probiotic treatment has a more marked influence on the microbiota composition with respect to the short intervention. An opposite trend was observed for Prevotellaceae, which also belong to Bacteroidales that decreased, although not significantly, in the group receiving the longer probiotic intervention. The decrease cannot probably be ascribed to the probiotic intervention but to a general progression of the disease, as already observed in PD [3]*.*

The administered *Lactobacillus* strains may have acted against some Clostridales families (Christensellaceae, Clostridiales vadin BB60 group, Clostridiaceae, and Lachnospiraceae) in agreement with a number of studies describing the effectiveness of *Lactobacillus* strains against *Clostridium* species, especially against cytotoxicity and adhesion to the gut epithelium of some *Clostridium* strains [77–79]*.* Through direct and indirect actions, co-administration of probiotics could prevent *Clostridium difficile* infection [80, 81].

Differently, Ruminoccoccaceae showed a fluctuating trend increasing after 3 months and then decreasing in the following 3 months regardless the treatment. As already reported, this is one of the main microbial groups showing differences between controls and diseased subjects [12]*.*

The Verrucomicrobiaceae decrease, although not significant, only in group A, is in agreement with literature studies related to probiotic interventions against *Clostridium* infection in mice, which showed that Verrucomicrobiaceae are particularly sensitive to *Bifidobacterium* and *Lactobacillus* supplementation [82], and with studies on PD [3, 83]*.* Therefore, the increase of this microbial group may be implicated in the pathogenesis of neurological diseases, as PD and ALS, and the probiotic formula experimented in this study may be effective in contrasting the variation of Verrucomicrobiaceae if administered for 6 continuous months.

Furthermore, 6 months probiotic administration may have contrasted some microbial groups potentially harmful for the host, such as *E. coli*, *Clostridium* cluster I, and Enterobacteriaceae, as shown by qPCR analysis. The counts of total yeasts also decreased upon probiotic administration, thus showing that the probiotic formulation had no stimulating effects on this microbial group.

Some changes were observed considering only time as the variable, and they can be associated with the progression of the disease. Bacteroidetes and related families were the microbial groups that changed the most with time. The significant increase after 3 months (T1) from baseline (T0) may represent a protective mechanism to counteract neurotoxicity, due to multiple functions, such as the stimulation of T cell-mediated responses, butyrate production, and bile acid, toxic, and/or mutagenic compound metabolism [84]*.*

The decrease of Cyanobacteria in both groups is not statistically significant. Since a positive correlation between Tenericutes and crude fiber digestibility has been shown in pigs [85], the significant decrease of this phylum after 3 months (T1) from baseline (T0) may be associated with a reduced fiber digestion and, therefore, a poorer nutritional status with the disease progression.

In addition, the reduction of α-diversity, calculated as observed OTU, was more related to the disease progression rather than to the treatment. α-diversity was also significantly lower in ALS patients with spinal onset at T2 in comparison with controls. Both the progression and the kind of onset may therefore affect the intra-individual biodiversity. Considering that the β-diversity changed upon the probiotic administration and that it was different in both treatments at T2 with respect to the baseline, it can be assumed that the duration of the probiotic treatment influenced the microbiota inter-individual biodiversity. However, in spite of these relevant changes, none of the interventions brought the gut microbiota biodiversity of ALS patients closer to that of controls and influenced the progression of the disease.

This is the first study that clearly shows the modifications of gut microbiota composition in ALS patients by applying novel and very rigorous methodologies. This approach can be applied in larger clinical studies incorporating different genetic and phenotypical disease variants in order to characterize the microbiota changes as a novel biomarker of the disease. Moreover, our study represents a preliminary clinic probiotic application in ALS patients, a field of study that relies only on very few works mainly performed in animal models. Our study suggests that some effects can be obtained in contrasting potentially pathogenic microbial groups and, thus, poses the bases for a microbial strategy to ameliorate the health status of the gut. The knowledge derived from this study can be applied in multicentric studies, creating a network involving a consistent number of ALS Centres to test novel microbial strategies for attenuating the ALS phenotype and progression. However, our study has limitations. Firstly, twenty patients did not complete the study and one died; therefore, the restricted group size at T1 and T2 hampered the statistical power of sequencing data. Secondly, the intra-individual variability both in patients and controls, due to different lifestyles, has not been considered in the study. Thirdly, the patients were recruited according to strict inclusion criteria aimed at the clinical trial; hence, an analysis of phenotypic subgroups is not feasible.

## Conclusions

The results that we obtained, besides increasing knowledge on the gut microbiota of ALS patients, show that ALS is associated to variations in some gut microbial components with respect to controls also in patients with low disability and full vital functions. We have demonstrated in this study that the gut microbiota composition changes during the course of the disease as demonstrated by the significant fluctuations of some microbial groups during the follow-up. Interestingly, an unbalance between potentially protective microbial groups, such as members of Bacteroidales, and other with potential neurotoxic or pro-inflammatory activity, such as Cyanobacteria, has been shown. Our study poses the bases for larger clinical studies to characterize the microbiota changes as a novel ALS biomarker and to test new microbial strategy to ameliorate the health status of the gut.

## Supplementary information

**Additional file 1: Figure S1.** OTUs distribution in the control-diseased dataset for the most representative phyla. a) OTUs distribution for Bacteroidetes, Firmicutes, Actinobacteria and Verrucomicrobia. b) OTUs distribution for Cyanobacteria. **Figure S2.** OTUs distribution in the control-diseased dataset for the significant families. a) OTUs distribution for Gastranaerophilales; D_4__uncultured bacterium and Clostridiaceae 1. b) OTUs distribution for Clostridiales; Ambiguous_taxa and Clostridiales;D_4__Family XI. **Figure S3**. Gut bacterial families and genera characteristics at baseline. a) Relative abundance of microbial groups at family level in the control group (C) and diseased group (D); families with a relative abundance less than 0.002 are omitted for the sake of clarity. b) Relative abundance of microbial groups at genus level in the control group (C) and diseased group (D); genera with a relative abundance less than 0.003 are omitted for the sake of clarity. **Figure S4.** Fecal yeast qPCR counts during the intervention. The graphic was conceived as mean plot reporting also the standard errors. **Figure S5.** OTUs distribution in ALS patient’s dataset for significant families. a) OTUs distribution for Bacteroidales; other among the ALS patients grouped for different timepoints and not considering the type of treatment. b) OTUs distribution for Baceroidaceae and Rikenellaceae among ALS patients considering the time and the different treatments (Group A and Group B). **Table S1.** Absolute abundance expressed as means of the number of OTUs for phyla and families that were significantly different between control (C) and diseased subjects (D) at the baseline. **Table S2.** Absolute abundance expressed as means of the number of OTUs for phyla and families that were significantly different among ALS patients during the study. “*P*_corr_” corresponds to the adjusted *P*-value for the comparisons performed. **Table S3.** DNA concentration and mean counts of the analyzed microbial groups by qPCR in stool samples of ALS patients during the intervention. **Table S4.** Relative abundance of the main families for each group of subjects during the intervention.

## Data Availability

The datasets used and/or analyzed during the current study are available from the corresponding author on reasonable request.

## Abbreviations

AD: Alzheimer’s disease
AE: Adverse event
ALS: Amyotrophic lateral sclerosis
ALSFRS-R: ALS Functional Rating Scale–Revised
ANOVA: Analysis of variance
BDNF: Brain-derived neurotrophic factor
BMAA: Amino acid β-methylamino-l-alanine
BMI: Body mass index
C9Orf72: Chromosome 9 open reading frame 72
CE: Comitato Etico
CFU: Colony-forming unit
CNS: Central nervous system
DNA: Deoxyribonucleic acid
F/B: Firmicutes/Bacteroidetes
FVC%: Force vital capacity percentage
FUS: Fused in sarcoma
HFRS: High Functional Rating Scale
LFRS: Low Functional Rating Scale
IBS: Inflammatory bowel syndrome
OTU: Operational taxonomic unit
NGS: Next generation sequencing
PCR: Polymerase chain reaction
PCR-DGGE: PCR-Denaturing Gradient Gel Electrophoresis
qPCR: Quantitative PCR
PD: Parkinson’s disease
PDC: PD and dementia
rrnDB: Ribosomal RNA Operon Copy Number Database
SCFA: Short chain fatty acid
SD: Standard deviation
SOD1: Superoxide dismutase-1
TARDP: TAR DNA-binding protein 43
T0: Time 0 (baseline)
T1: Time 1
T2: Time 2
UPGMA: Unweighted pair-group method with the arithmetic average
