# Supplementary material for: A prospective longitudinal study on the microbiota composition in amyotrophic lateral sclerosis
Source: BMC Med. 2020 Jun 17;18:153. doi: 10.1186/s12916-020-01607-9 (PMC7298784; doi:10.1186/s12916-020-01607-9)
Supplement: Supplementary file 1 — Additional file 1: Figure S1. OTUs distribution in the control-diseased dataset for the most representative phyla. a) OTUs distribution for Bacteroidetes, Firmicutes, Actinobacteria and Verrucomicrobia. b) OTUs distribution for Cyanobacteria. Figure S2. OTUs distribution in the control-diseased dataset for the significant families. a) OTUs distribution for Gastranaerophilales; D_4__uncultured bacterium and Clostridiaceae 1. b) OTUs distribution for Clostridiales; Ambiguous_taxa and Clostridiales;D_4__Family XI. Figure S3. Gut bacterial families and genera characteristics at baseline. a) Relative abundance of microbial groups at family level in the control group (C) and diseased group (D); families with a relative abundance less than 0.002 are omitted for the sake of clarity. b) Relative abundance of microbial groups at genus level in the control group (C) and diseased group (D); genera with a relative abundance less than 0.003 are omitted for the sake of clarity. Figure S4. Fecal yeast qPCR counts during the intervention. The graphic was conceived as mean plot reporting also the standard errors. Figure S5. OTUs distribution in ALS patient’s dataset for significant families. a) OTUs distribution for Bacteroidales; other among the ALS patients grouped for different timepoints and not considering the type of treatment. b) OTUs distribution for Baceroidaceae and Rikenellaceae among ALS patients considering the time and the different treatments (Group A and Group B). Table S1. Absolute abundance expressed as means of the number of OTUs for phyla and families that were significantly different between control (C) and diseased subjects (D) at the baseline. Table S2. Absolute abundance expressed as means of the number of OTUs for phyla and families that were significantly different among ALS patients during the study. “Pcorr” corresponds to the adjusted P-value for the comparisons performed. Table S3. DNA concentration and mean counts of the analyzed microbial groups by qPCR [file 12916_2020_1607_MOESM1_ESM.docx]

**Additional file 1**

**Figures**

**
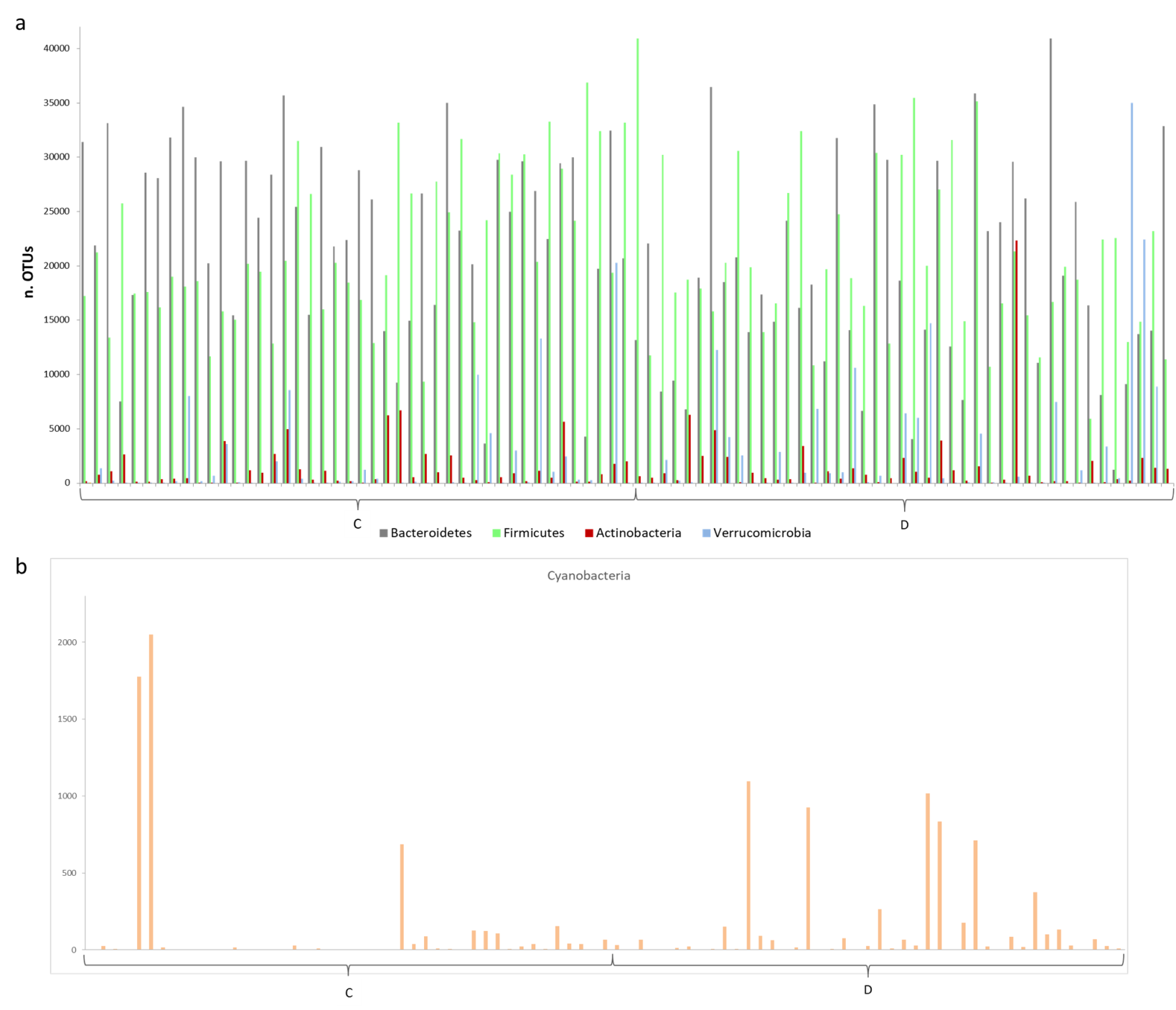
**

**Figure S1.** OTUs distribution in the control-diseased dataset for the most representative phyla. a) OTUs distribution for Bacteroidetes, Firmicutes, Actinobacteria and Verrucomicrobia. b) OTUs distribution for Cyanobacteria.


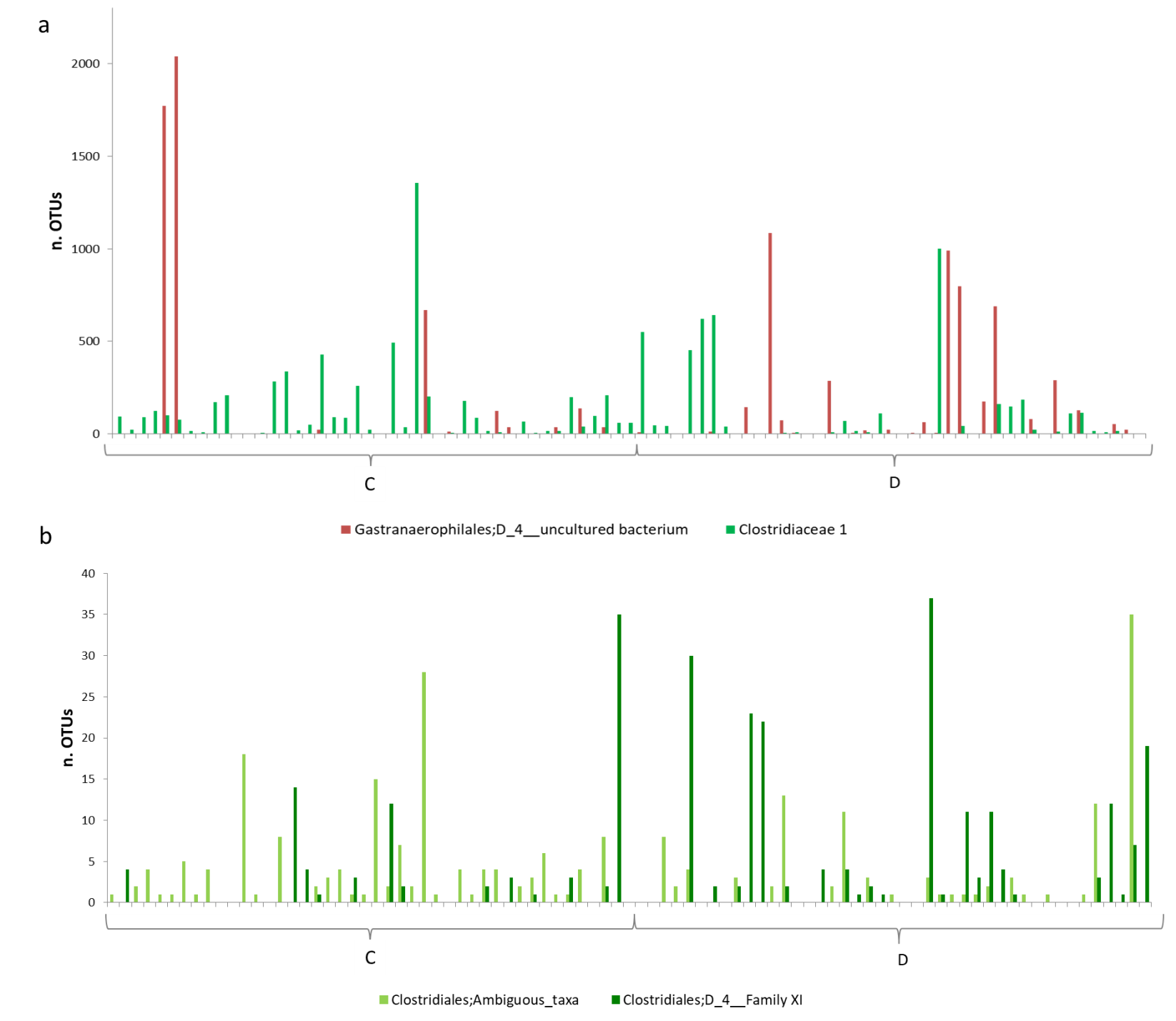


**Figure S2.** OTUs distribution in the control-diseased dataset for the significant families. a) OTUs distribution for Gastranaerophilales;D_4__uncultured bacterium and Clostridiaceae 1. b) OTUs distribution for Clostridiales;Ambiguous_taxa and Clostridiales;D_4__Family XI.

**
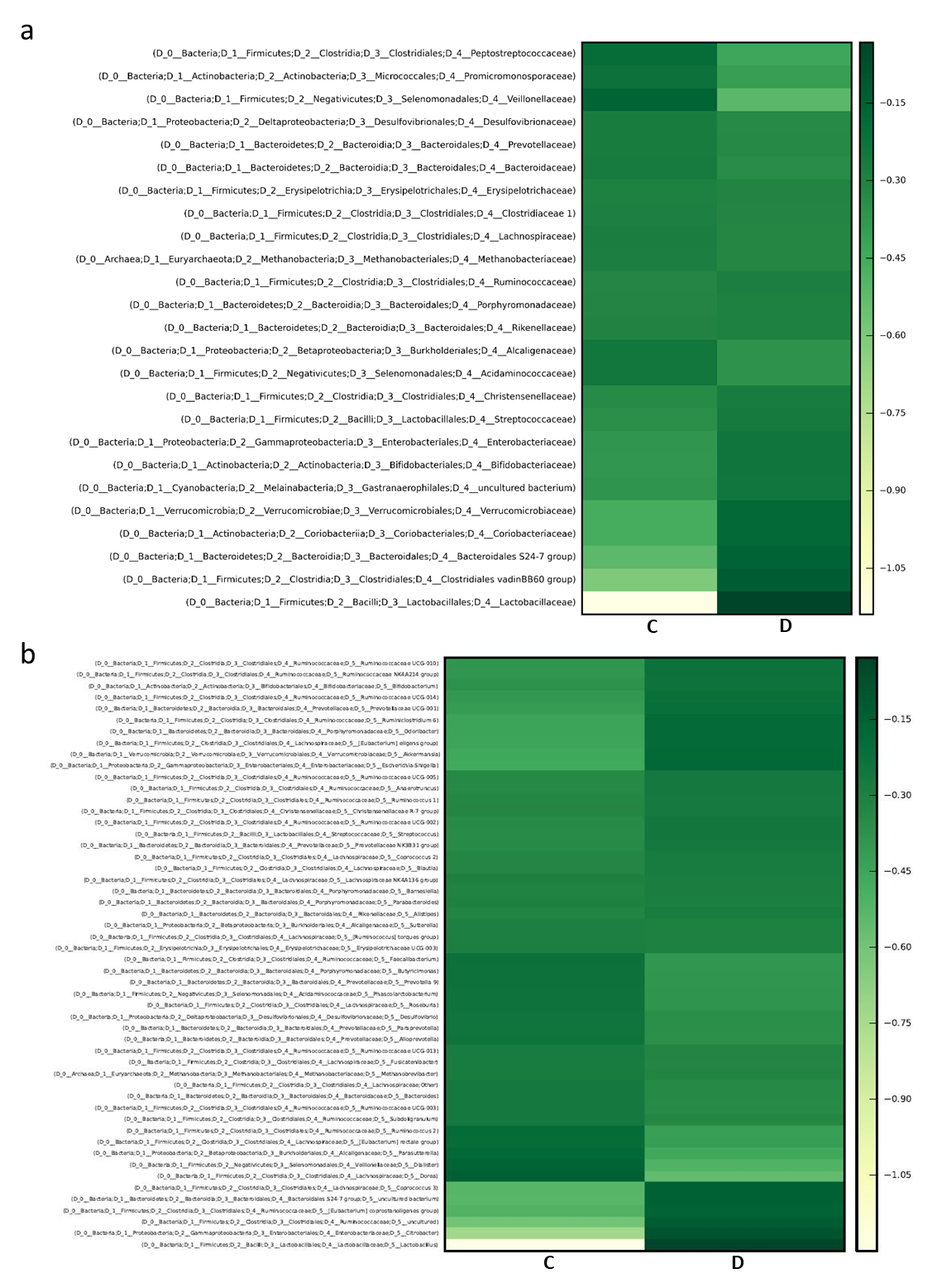
**

**Figure S3.** Gut bacterial families and genera characteristics at baseline. a**)** Relative abundance of microbial groups at family level in the control group (C) and diseased group (D); families with a relative abundance less than 0.002 are omitted for the sake of clarity. b) Relative abundance of microbial groups at genus level in the control group (C) and diseased group (D); genera with a relative abundance less than 0.003 are omitted for the sake of clarity.


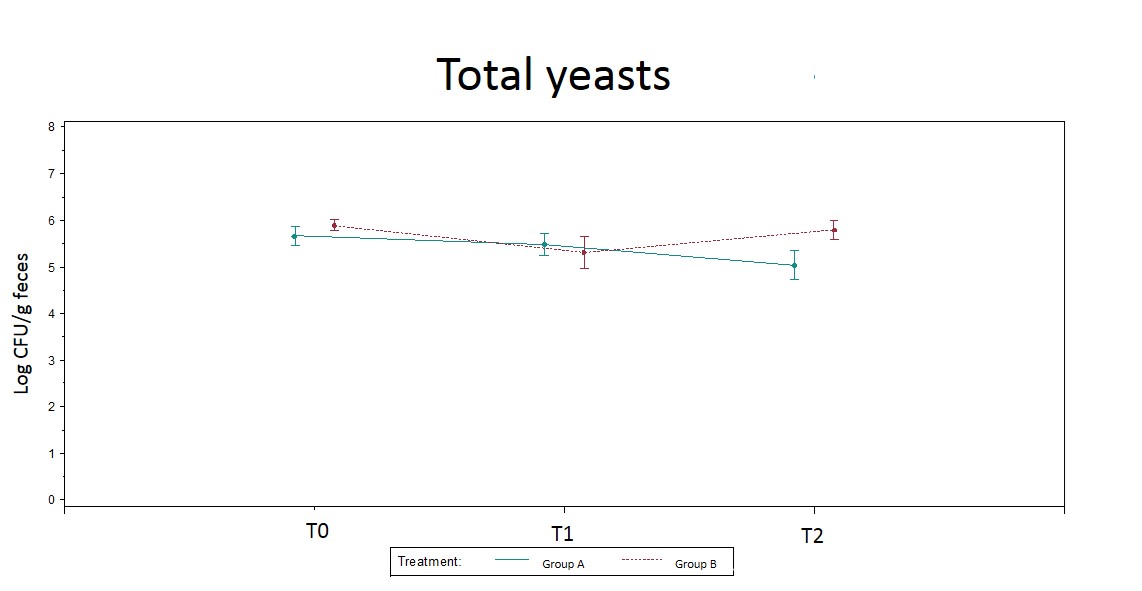


**Figure S4.** Fecal yeast qPCR counts during the intervention. The graphic was conceived as mean plot reporting also the standard errors.

**
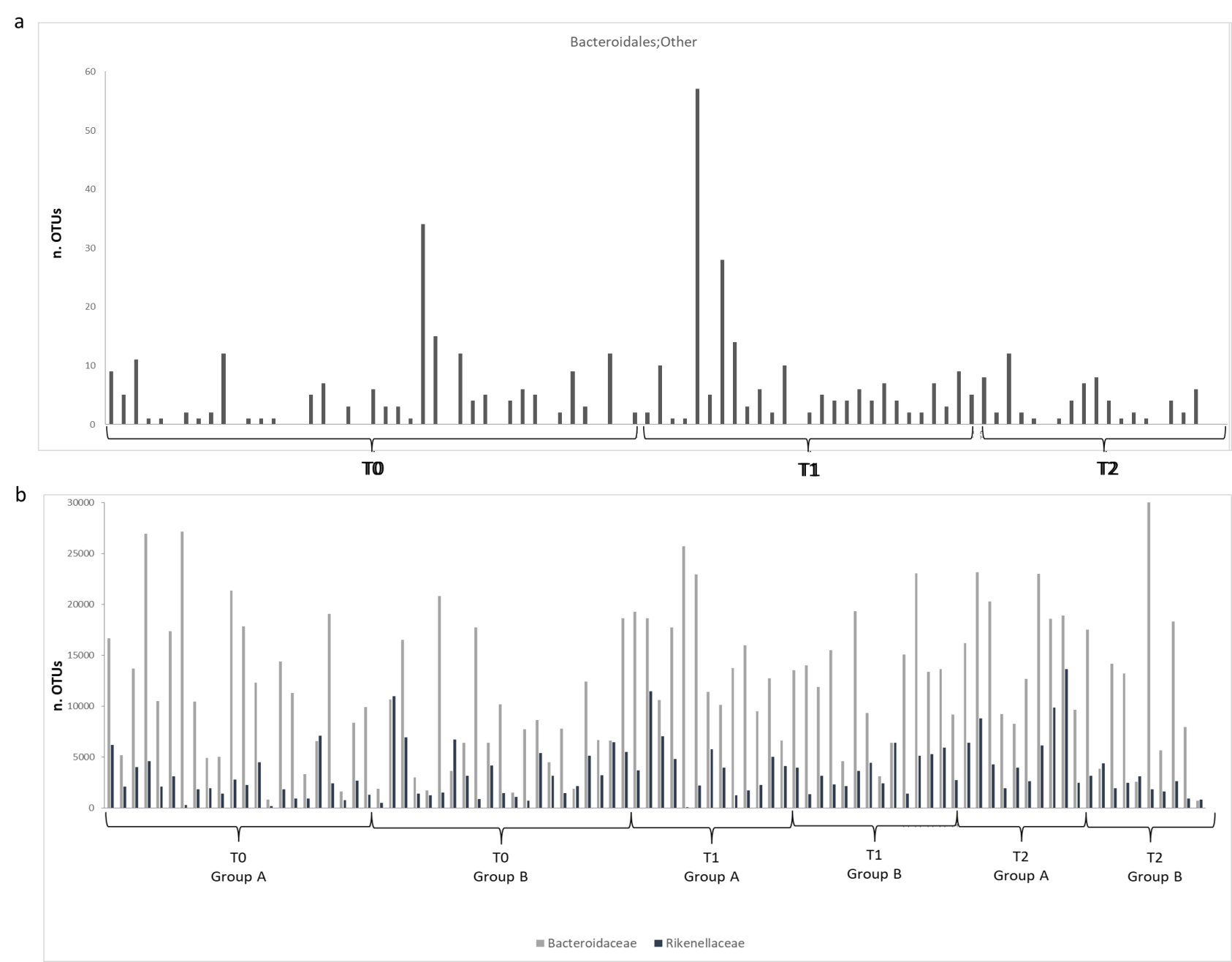
**

**Figure S5.** OTUs distribution in ALS patient dataset for significant familes. a) OTUs distribution for Bacteroidales;other among the ALS patients grouped for different timepoints and not considering the type of treatment. b) OTUs distribution for Baceroidaceae and Rikenellaceae among ALS patients considering the time and the different treatments (Group A and Group B).

**Tables**

**Table S1**. Absolute abundance expressed as means of the number of OTUs for phyla and families that were significantly different between controls (C) and diseased subjects (D) at the baseline.

| **OTUs** | **C** | **D** | ***P*-value** |
| --- | --- | --- | --- |
| **Phyla** |  |  |  |
| Cyanobacteria | 125.16±409.83 | 152.79±293.54 | 0.031 |
| **Families** |  |  |  |
| Gastranaerophilalaes; D_4__uncultured bacterium | 111.02±410.01 | 115.81±265.07 | 0.015 |
| Clostridiales; Ambiguous taxa | 3.41±5.33 | 2.58±6.00 | 0.025 |
| Clostridiaceae 1 | 128.73±222.39 | 104.35±216.81 | 0.005 |
| Clostridiales; D_4__Familiy XI | 1.95±5.85 | 4.72±8.74 | 0.024 |

**Table S2.** Absolute abundance expressed as means of the number of OTUs for phyla and familes that were significantly different among ALS patients during the study. “*P*_corr_” corresponds to the adjusted *P*-value for the comparisons performed.

| **OTUs** | **T0** | | **T1** | | **T2** | | ***P*-value** | ***P*_corr_** |
| --- | --- | --- | --- | --- | --- | --- | --- | --- |
| **Phyla** |  | |  | |  | |  |  |
| Tenericutes | 114.84±275.52 ^a^ | | 13.37±29.13 ^b^ | | 38.80±98.63 ^ab^ | | 0.004 | 0.012 |
| Bacteroidetes | 40598.51±21545.38 ^a^ | | 51729.74±19843.78 ^b^ | | 60875.10±28458.40 ^ab^ | | 0.011 | 0.034 |
| **Families** |  | |  | |  | |  |  |
| Bacteroidaceae | 10243.51±6944.05 ^a^ | | 13687.18±5523.66 ^b^ | | 13584.89±7939.45 ^ab^ | | 0.013 | 0.039 |
| Bacteroidales; other | 4.37±6.14 ^a^ | | 7.53±11.11 ^b^ | | 3±3.25 ^a^ | | 0.009  0.010 | 0.029  0.029 |
| **OTU** | **Group A** | **Group B** | **Group A** | **Group B** | **Group A** | **Group B** |  |  |
| Rikenellaceae | 2529.41±  1803.73 ^a^ | 3504.38±2749.63 ^ab^ | 4287.64±  2890.45 ^ab^ | 3611.64±  1650.76 ^ab^ | 5981.78±  4008.07 ^b^ | 2303.10±  1093.47 ^a^ | 0.001  0.004 | 0.011  0.039 |

**Table S3.** DNA concentration and mean counts of the analyzed microbial groups by qPCR in stool samples of ALS patients during the intervention.

|  | Time | Group A | Group B | | *P*-value | |  |
| --- | --- | --- | --- | --- | --- | --- | --- |
|  |  | Mean (SD) | | Mean (SD) | |  | |
| DNA | T0 | 153.58 (120.16) | | 157.27 (119.65) | | 0.91 | |
|  | T1 | 132.44 (70.37) | | 120.61 (56.88) | | 0.63 | |
|  | T2 | 90.99 (54.9) | | 116.5 (52.42) | | 0.25 | |
| Total bacteria | T0 | 10.33 (0.86) | | 10.38 (0.87) | | 0.91 | |
|  | T1 | 10.46 (0.71) | | 10.15 (1.13) | | 0.38 | |
|  | T2 | 10.45 (0.48) | | 10.67 (0.64) | | 0.34 | |
| *Lactobacillus* spp. | T0 | 5.4 (1.54) | | 5.48 (0.96) | | 0.54 | |
|  | T1 | 5.61 (1.34) | | 5.21 (1.27) | | 0.36 | |
|  | T2 | 5.33 (1.28) | | 5.47 (0.78) | | 0.94 | |
| *Bifidobacterium* spp. | T0 | 7.07 (1.82) | | 7.52 (1.43) | | 0.42 | |
|  | T1 | 7.73 (0.95) | | 7.47 (1.85) | | 0.98 | |
|  | T2 | 6.78 (1.91) | | 7.79 (0.89) | | 0.24 | |
| *E. coli* | T0 | 6.64 (1.17) | | 6.55 (1.11) | | 0.78 | |
|  | T1 | 6.04 (1.9) | | 6.23 (1.65) | | 0.77 | |
|  | T2 | 5.66 (1.95) | | 6.93 (0.92) | | **0.05** | |
| *Clostridium* cluster I | T0 | 5.53 (1.3) | | 5.88 (1.76) | | 0.27 | |
|  | T1 | 5.48 (1.41) | | 5.72 (1.36) | | 0.63 | |
|  | T2 | 4.97 (1.16) | | 5.61 (0.81) | | 0.12 | |
| Enterobacteriaceae | T0 | 8.55 (0.78) | | 8.48 (0.82) | | 0.76 | |
|  | T1 | 8.13 (1.83) | | 8.08 (1.97) | | 0.95 | |
|  | T2 | 7.71 (2.07) | | 8.75 (0.56) | | 0.11 | |
| Total Yeasts | T0 | 5.66 (1) | | 5.89 (0.6) | | 0.32 | |
|  | T1 | 5.48 (0.97) | | 5.32 (1.24) | | 0.84 | |
|  | T2 | 5.04 (1.09) | | 5.8 (0.7) | | **0.03** | |

DNA concentrations are expressed as ng DNA/200 mg of faeces and the mean counts as Log CFU/g of feces, the related *P*- value is reported. Bolded values indicate *P* < 0.05.

**Table S4.** Relative abundance of the main families for each group of subjects.

| Families | Controls | T0  Group A | T1  Group A | T2  Group A | T0  Group B | T1  Group B | T2  Group B |
| --- | --- | --- | --- | --- | --- | --- | --- |
| Methanobacteriaceae | 0.637 | 0.394 | 0.184 | 0.804 | 0.782 | 1.572 | 0.517 |
| Actinomycetaceae | 0.009 | 0.009 | 0.006 | 0.015 | 0.011 | 0.010 | 0.009 |
| Bifidobacteriaceae | 2.073 | 1.191 | 2.191 | 1.257 | 4.486 | 4.147 | 5.817 |
| Promicromonosporaceae | 0.287 | 0.169 | 0.152 | 0.452 | 0.208 | 0.2583 | 0.175 |
| Coriobacteriaceae | 0.223 | 0.378 | 0.439 | 0.989 | 0.476 | 0.498 | 0.722 |
| Bacteroidaceae | 24.109 | 24.114 | 29.404 | 32.328 | 16.085 | 25.195 | 25.240 |
| Bacteroidales S24-7 group | 1.038 | 1.328 | 0.590 | 0.246 | 3.451 | 1.742 | 2.498 |
| Porphyromonadaceae | 5.318 | 5.262 | 8.682 | 8.685 | 5.820 | 7.763 | 4.580 |
| Prevotellaceae | 8.086 | 5.896 | 3.682 | 1.910 | 8.310 | 9.881 | 6.514 |
| Rikenellaceae | 5.965 | 4.851 | 8.732 | 12.249 | 7.22 | 7.144 | 4.823 |
| Bacteroidales; uncultured bacterium | 0.080 | 0.094 | 0.074 | 0.048 | 0.164 | 0.388 | 0.177 |
| Bacteroidales;Other | 0.007 | 0.007 | 0.021 | 0.007 | 0.009 | 0.010 | 0.004 |
| Gastranaerophilales;uncultured bacterium | 0.173 | 0.351 | 0.019 | 0.102 | 0.114 | 0.088 | 0.013 |
| Lactobacillaceae | 0.048 | 0.142 | 0.068 | 0.034 | 1.091 | 0.036 | 0.045 |
| Streptococcaceae | 0.362 | 0.609 | 0.266 | 0.212 | 0.286 | 0.213 | 0.217 |
| Christensenellaceae | 2.462 | 2.816 | 2.016 | 1.225 | 2.908 | 3.118 | 2.393 |
| Clostridiaceae 1 | 0.248 | 0.163 | 0.108 | 0.025 | 0.307 | 0.200 | 0.070 |
| Clostridiales vadinBB60 group | 0.104 | 0.265 | 0.198 | 0.061 | 0.365 | 0.130 | 0.270 |
| Defluviitaleaceae | 0.015 | 0.012 | 0.024 | 0.012 | 0.032 | 0.019 | 0.011 |
| Clostridiales;Family XIII | 0.146 | 0.225 | 0.533 | 0.212 | 0.156 | 0.195 | 0.104 |
| Lachnospiraceae | 11.484 | 10.518 | 9.020 | 7.588 | 10.443 | 7.980 | 9.167 |
| Peptococcaceae | 0.114 | 0.111 | 0.105 | 0.105 | 0.139 | 0.256 | 0.108 |
| Peptostreptococcaceae | 0.443 | 0.250 | 0.109 | 0.104 | 0.266 | 0.153 | 0.092 |
| Ruminococcaceae | 20.908 | 23.617 | 18.248 | 22.755 | 21.137 | 17.813 | 21.021 |
| Clostridiales;Other | 0.018 | 0.012 | 0.015 | 0.008 | 0.019 | 0.019 | 0.012 |
| Thermoanaerobacteraceae | 0.050 | 0.039 | 0.065 | 0.040 | 0.031 | 0.017 | 0.022 |
| Erysipelotrichaceae | 0.887 | 1.102 | 0.310 | 0.558 | 0.637 | 0.515 | 0.402 |
| Acidaminococcaceae | 2.265 | 1.643 | 1.666 | 1.230 | 1.836 | 2.237 | 2.085 |
| Veillonellaceae | 4.250 | 1.347 | 2.837 | 1.448 | 2.390 | 0.886 | 1.206 |
| Fusobacteriaceae | 0.002 | 0.007 | 0.011 | 0.016 | 0.005 | 0.007 | 0.029 |
| Victivallaceae | 0.005 | 0.015 | 0.002 | 0.011 | 0.014 | 0.007 | 0.025 |
| Rhodospirillaceae | 0.129 | 0.304 | 0.474 | 0.506 | 0.056 | 0.227 | 0.202 |
| Oxalobacteraceae | 0.020 | 0.044 | 0.016 | 0.023 | 0.021 | 0.047 | 0.058 |
| Desulfovibrionaceae | 0.680 | 0.817 | 0.543 | 0.525 | 0.368 | 0.445 | 0.526 |
| Enterobacteriaceae | 1.717 | 1.656 | 2.463 | 0.752 | 3.025 | 1.243 | 3.190 |
| Pasteurellaceae | 0.345 | 0.029 | 0.026 | 0.008 | 0.036 | 0.027 | 0.032 |
| Synergistaceae | 0.128 | 0.327 | 0.304 | 0.159 | 0.071 | 0.118 | 0.398 |
| Mollicutes RF9; uncultured bacterium | 0.016 | 0.035 | 0.002 | 0.022 | 0.075 | 0.004 | 0.029 |
| Mollicutes; NB1-n;Ambiguous taxa | 0.012 | 0.012 | 0.002 | 0.004 | 0.049 | 0.008 | 0.005 |
| Opitutae vadinHA64; uncultured bacterium | 0.022 | 0.042 | 0.006 | 0.001 | 0.051 | 0.039 | 0.055 |
| Verrucomicrobiaceae | 3.557 | 7.937 | 4.409 | 1.067 | 5.562 | 3.643 | 5.994 |

Data are expressed as percentage of relative abundance; families with a relative abundance less than 0.001% are omitted for the sake of clarity
